# Ending preventable maternal mortality: phase II of a multi-step process to develop a monitoring framework, 2016–2030

**DOI:** 10.1186/s12884-018-1763-8

**Published:** 2018-06-25

**Authors:** R. Rima Jolivet, Allisyn C. Moran, Meaghan O’Connor, Doris Chou, Neelam Bhardwaj, Holly Newby, Jennifer Requejo, Marta Schaaf, Lale Say, Ana Langer

**Affiliations:** 1 Maternal Health Task Force, Women & Health Initiative, Harvard T.H. Chan School of Public Health, 651 Huntington Avenue, Boston, MA 02115 USA; 2 Department of Maternal, Newborn, Child and Adolescent Health, World Health Organization, 20, Avenue Appia CH-1211, 27 Geneva, Switzerland; 3 Department of Reproductive Health and Research, World Health Organization, 20 Avenue Appia, 1211 Geneva, Switzerland; 4 United Nations Population Fund, 605 3rd Ave, New York, NY 10158 USA; 5 Independent Consultant, Stockholm, Sweden; 6 Johns Hopkins University, 615 N Wolfe St, Baltimore, MD 21205 USA; 7 Averting Maternal Death & Disability Program (AMDD), Heilbrunn Department of Population and Family Health, Mailman School of Public Health, Columbia University, 60 Haven Avenue, B3, New York, NY 10032 USA

**Keywords:** Maternal health, Maternal mortality, Indicators, Monitoring, Social determinants of health

## Abstract

**Background:**

In February 2015, the World Health Organization (WHO) released “Strategies toward ending preventable maternal mortality (EPMM)” (EPMM Strategies), a direction-setting report outlining global targets and strategies for reducing maternal mortality in the Sustainable Development Goal (SDG) period. In May 2015, the EPMM Working Group outlined a plan to develop a comprehensive monitoring framework to track progress toward the achievement of these targets and priorities. This monitoring framework was developed in two phases. Phase I, which focused on identifying indicators related to the proximal causes of maternal mortality, was completed in October 2015. This paper describes the process and results of Phase II, which was completed in November 2016 and aimed to build consensus on a set of indicators that capture information on the social, political, and economic determinants of maternal health and mortality.

**Findings:**

A total of 150 experts from more than 78 organizations worldwide participated in this second phase of the process to develop a comprehensive monitoring framework for EPMM. The experts considered a total of 118 indicators grouped into the 11 key themes outlined in the EPMM report, ultimately reaching consensus on a set of 25 indicators, five equity stratifiers, and one transparency stratifier.

**Conclusion:**

The indicators identified in Phase II will be used along with the Phase I indicators to monitor progress towards ending preventable maternal deaths. Together, they provide a means for monitoring not only the essential clinical interventions needed to save lives but also the equally important political, social, economic and health system determinants of maternal health and survival. These distal factors are essential to creating the enabling environment and high-performing health systems needed to ensure high-quality clinical care at the point of service for every woman, her fetus and newborn. They complement and support other monitoring efforts, in particular the “Survive, Thrive, and Transform” agenda laid out by the Global Strategy for Women’s, Children’s and Adolescents’ Health (2016-2030) and the SDG3 global target on maternal mortality.

**Electronic supplementary material:**

The online version of this article (10.1186/s12884-018-1763-8) contains supplementary material, which is available to authorized users.

## Background

Global maternal mortality remains unacceptably high, with an estimated 303,000 women dying each year as a result of pregnancy and childbirth-related complications [1]. Although maternal deaths worldwide declined by 44% between 1990 and 2015, this achievement fell far short of the 75% reduction targeted by Millennium Development Goal 5a [1, 2]. As of 2015, 25 countries still had a maternal mortality ratio (MMR) of 420 per 100,000 live births or greater [1, 2]. Furthermore, the staggering 80-fold difference in the estimated lifetime risk of maternal mortality in low-income countries, as compared to high-income countries, points to the persistence of profound inequality that must be addressed.

The reasons for lack of progress are complex and multifactorial. A recent series on maternal health points out increasing diversification in the causes of maternal mortality and morbidity that health systems are ill equipped to address. Disparities in access to care especially for vulnerable populations, poor quality of available care, grave deficiencies in health system infrastructure and workforce, and the impact of economic, political, socio-demographic and environmental factors all contribute significantly to the risk of poor maternal health outcomes and impede progress toward reduction of mortality and morbidity [3–8].

To address the lack of steady progress across all countries and reassert the importance of the unfinished agenda of reducing maternal mortality in the Sustainable Development Goal (SDG) era, the World Health Organization (WHO) released a direction setting report entitled “Strategies toward ending preventable maternal mortality (EPMM)” (EPMM Strategies) in February 2015 [9]. The report, developed through extensive consultations led by the EPMM Working Group^1^, outlines targets and strategies for reducing preventable maternal deaths worldwide. The targets are both national and global (Table 1). The global target was adopted by the SDG framework.

**Table 1:** National and global maternal mortality rate targets

**Global target for maternal mortality (SDG Target 3.1)** By 2030, reduce the global maternal mortality ratio to less than 70 deaths per 100,000 live births. **National targets for maternal mortality** By 2030, all countries should reduce their maternal mortality ratios by at least two-thirds from 2010 baseline; countries with the highest maternal mortality burdens will need a further reduction. AND By 2030, no country should have a maternal mortality ratio greater than 140 deaths per 100,000 live births, a number twice the global target.	

The strategies outlined in the report are exemplified by 11 key themes that are grounded in a human rights-based approach to health and focus heavily on the principles of equity and non-discrimination, transparency, participation, and accountability to ensure that reproductive, maternal, and newborn health care is available, accessible, and acceptable to all who need it (Table 2). The themes point to the need to assess and address not only the most proximal causes of maternal death, but also the broad range of more distal systemic and social determinants of maternal health and survival.

**Table 2:** EPMM Key Themes

Guiding Principles	1. Empower women, girls, families and communities
	2. Integrate maternal and newborn health, protect and support the mother-baby dyad
	3. Prioritize country ownership, leadership, and supportive legal, regulatory and financial frameworks
	4. Apply a human-rights framework to ensure that high-quality reproductive, maternal, and newborn health care is available, accessible and acceptable to all who need it
Cross-cutting Actions	5. Improve metrics, measurement systems, and data quality
	6. Prioritize adequate resources and effective health care financing
Five Strategic Objectives	7. Address inequities in access to and quality of sexual, reproductive, maternal and newborn healthcare
	8. Ensure universal health coverage for comprehensive sexual, reproductive, maternal, and newborn healthcare
	9. Address all causes of maternal mortality, reproductive and maternal morbidities and related disabilities
	10. Strengthen health systems to respond to the needs and priorities of women and girls
	11. Ensure accountability in order to improve quality of care and equity

To support attaining the ambitious SDG MMR target and the maternal health-related aims embedded in the three pillars (“Survive Thrive, and Transform”) of the UN Secretary General’s Global Strategy for Women’s, Children’s and Adolescents’ Health (2016-2030) (Global Strategy), it is essential for all countries to track and monitor progress in the areas outlined in the key themes [10]. In May 2015, the EPMM Working Group outlined a two-phased plan to develop a comprehensive monitoring framework to track national and global progress towards improving maternal health and survival. The first phase (Phase I) involved selecting measures to address the most proximal causes of maternal mortality while the second phase (Phase II) focused on identifying indicators to address the distal causes (social, political, and economic determinants) of maternal mortality.

Phase I, completed in October 2015, resulted in a set of 12 core metrics for global monitoring and national reporting by all countries, along with four priority areas in which further work is required to develop other, much needed indicators. This process was described by Moran et al. (2016) [11]. The set of EPMM core metrics from Phase I fed into the development of two key monitoring initiatives: the Indicator and Monitoring Framework for the Global Strategy (published in February 2016), and the Countdown to 2030’s selection of coverage and demographic indicators for its country profiles [12]. To date, global initiatives for tracking progress in health have focused primarily on coverage and impact measures for tracking key clinical interventions. For the majority of the maternal health coverage and impact indicators identified in Phase I, there is a history of standardized global and national-level monitoring. Thus, a major achievement of that effort was to achieve consensus on a priority set for global monitoring and national reporting by all countries.

**Fig. 1:**
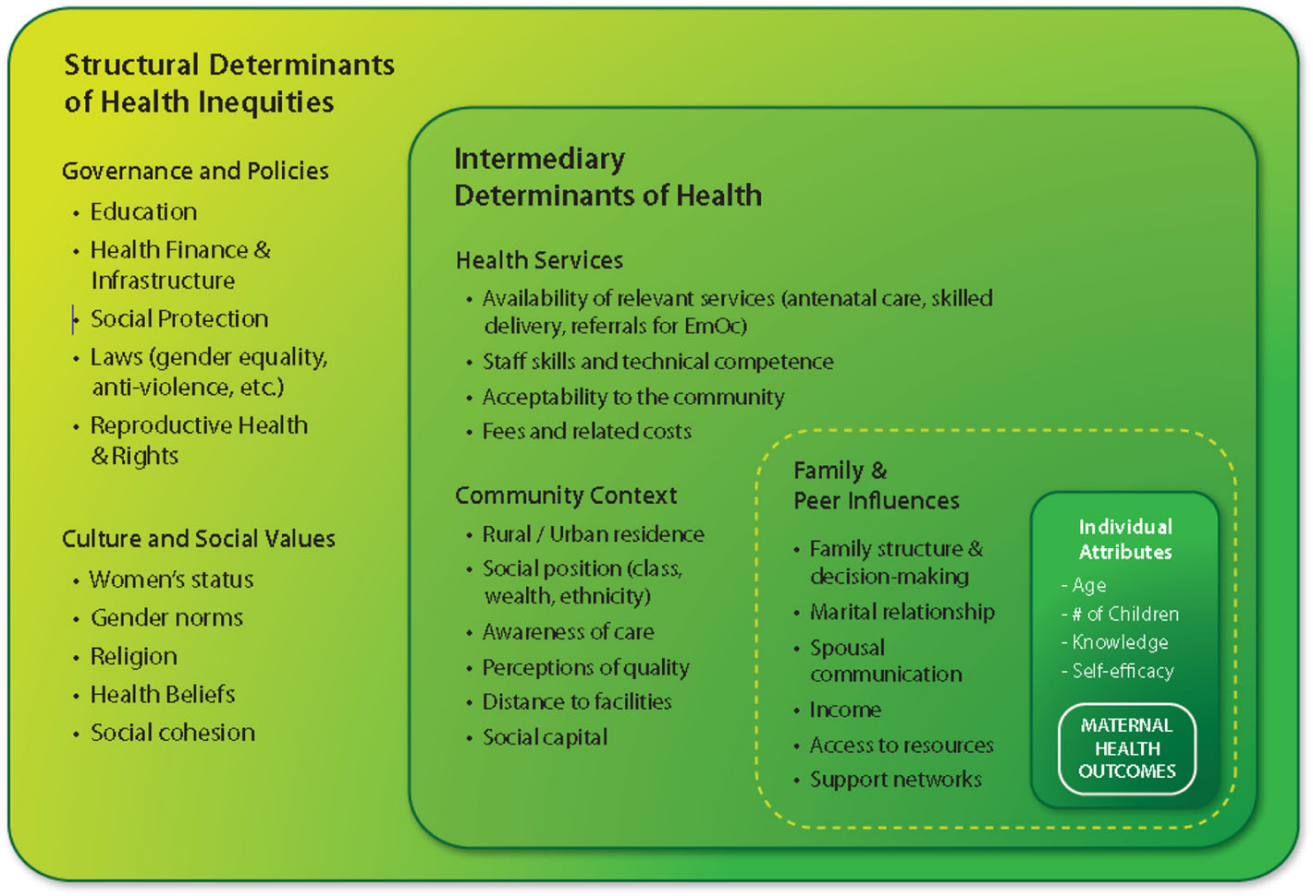
Structural Determinants of Health Inequities

This paper, however, describes the process and results of Phase II, which was completed in November 2016 and focused on identifying indicators that address the broad social, political, and economic determinants of maternal health as outlined by the 11 key themes in the EPMM Strategies. Because such distal determinants are risk factors for poor maternal health outcomes as well as elements of the enabling environment necessary for effective interventions, addressing these distal determinants is a critical factor for successfully improving maternal health and survival [13, 14]. Furthermore, recognition of the importance of these more distal determinants of health continues to grow, and experience demonstrates that when technical strategies aiming to address maternal health risks do not include attention to the broader supportive context, those strategies are unlikely to succeed [3, 14] (Fig. 1).

Expanding the pursuit of optimal health from a simply technical issue to a complex social phenomenon reframes health improvement as a matter of social justice, and indeed, in recent years, there has been an increase in attention to maternal mortality reduction as a human rights issue. Thus, the effort in Phase II to identify a set of supplementary indicators focused on the distal determinants of maternal health and survival serves to support the Phase I work and to round out a comprehensive monitoring framework for EPMM based on a human rights-based, social determinants approach to maternal health.

## Methods

The Maternal Health Task Force (MHTF), the U.S. Agency for International Development (USAID), and WHO led the technical work to identify, evaluate, and prioritize indicators on distal determinants of maternal health and survival to reach consensus on a minimum set for national and global level monitoring and reporting. The process is described in detail below.

A steering committee (composed of the authors of this paper) was formed to plan and guide the indicator selection process. The steering committee was guided by the following research question:

“Working within the specific context of maternal health, and using the priority recommendations outlined in the EPMM Strategies report, what are the 1-3 strongest indicators available for each of the 11 EPMM key themes from the EPMM Strategies report that can, together, help track progress towards addressing the social, political, and economic determinants of maternal health and survival?”

The steering committee conducted a review of selection criteria used to evaluate indicators in other measure development efforts, comparing criteria from a number of sources [12, 15–18]. The resulting set of selection criteria were then used throughout the process (Table 3).

**Table 3:** Phase II Indicator Selection Criteria

Relevance	• Indicator directly supports EPMM strategies for reducing preventable maternal mortality
	• There is evidence that what the indicator measures is significantly associated with improved maternal health and survival
Importance	• Indicator resonates, and is valuable to decision makers and stakeholders
	• Indicator “makes a difference” for improving maternal health and survival across countries and contexts
Interpretability & Usefulness	• There is good/strong evidence to support the process, or the outcome
	• Results point to areas for improvement and can advance strategic planning, policy or programming at different levels of the system
Validity	• Indicator measures what it is supposed to measure
	• Indicator has been field-tested and used
	• Indicator makes sense logically and scientifically
Feasibility & Data Availability	• Based on the best available data of acceptable quality
	• Data can be obtained with reasonable and affordable efforts in timely manner
	• Data does not overly increase reporting burden on countries
Harmonization	• Indicator strengthens or compliments existing efforts
	• Indicator is recommended and being used by leading experts and organizations
	• Indicator lacks redundancy and does not measure something already captured under other indicators

**Fig. 2:**
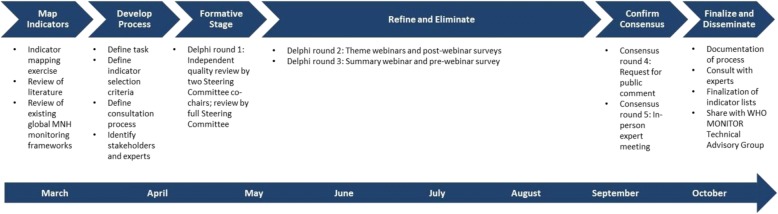
Phase II Process to Develop a Monitoring Framework for EPMM

The Phase II indicators and stratifiers were identified through a rigorous, iterative process that included multiple rounds of expert review and consultations conducted over a period of eight months (Fig. 2). Guided by the priority recommendations for each of the 11 key themes in the EPMM Strategies report, the formative stage of the process, round 1, began with a mapping exercise to identify potential indicators and, where applicable, stratifiers for each theme. The priority recommendations for each key theme from the EPMM Strategies report were summarized and used to guide the search for relevant indicators for that theme. Additional file 1 displays the priority recommendations, as well as the indicators mapped, for each key theme.

Indicator mapping included a thorough review of the peer-reviewed and gray literature to capture all indicators currently in use, under development, and not currently in routine use. The search identified an average of 33 indicators for each theme. It should be noted that the search for the theme focused on improving equity in access to and quality of maternal and newborn health care also included stratifiers, because an essential approach to tracking equity is the disaggregation of data by specific factors (such as wealth, sex, or age) that place some people at a social disadvantage. Such stratifiers allow measurement of an indicator’s performance for subpopulations relative to the total population that is captured in the indicator’s denominator.

All indicators (and stratifiers) were independently reviewed for quality by steering committee co-chairs (AM and RJ), and indicators that clearly did not meet the criteria were eliminated. The full steering committee reviewed the remaining indicators, making further eliminations. Differences of opinion were resolved through discussion. During round 1, an average of 11 potential indicators per EPMM key theme (plus an additional seven stratifiers for the equity-focused theme) was selected by the steering committee for advancement to the second round of expert and stakeholder review and consideration.

In the next two rounds, a modified Delphi method was used to systematically evaluate and rank order potential indicators, with the goal of identifying up to three of the strongest available for monitoring each of the 11 key themes [19]. Round 2 of review included a series of 11 webinars and 11 quantitative surveys (one webinar and one follow-up survey per key theme). Each theme’s webinar was attended by a panel of experts who were selected to reflect technical, policy, and in-country monitoring expertise in the relevant subject area. The steering committee decided on an ideal panel size of 10-15 individuals based on the literature regarding suggested Delphi method group size [20, 21]. For each webinar an average of 62 experts were invited, and an average of 18 experts ultimately participated.

The webinars were facilitated by a steering committee co-chair (RJ) and panelists debated the merits of each potential indicator against the selection criteria to agree on the three strongest indicators for that theme. Following each webinar, a quantitative survey was sent to the full invitation list, including those who were unable to join the webinar. In the survey, participants were asked to score each indicator for quality using the predetermined selection criteria and to rank up to three indicators as the strongest for monitoring progress towards the specific theme. For all 11 themes, the results of the individual quantitative surveys confirmed the consensus reached during the webinar discussion. In round 2, a total of 118 indicators were evaluated, 50 indicators were eliminated, and 38 indicators were selected for potential inclusion in the final set of EPMM Phase II indicators.

Round 3 of the modified Delphi method consisted of a final, summary webinar and corresponding online quantitative survey. In this round, the indicators that were ranked among the top three during each of the 11 thematic webinars were evaluated as a full set and further prioritized. This summary webinar included a total of 18 experts who participated in past webinars as well as additional key stakeholders. The panel reviewed and rank-ordered the 38 indicators that emerged from round 2, ultimately eliminating seven indicators during this round and bringing the total number of indicators in the draft set of EPMM Phase II core indicators to 31.

Shifting from a focus on elimination, the fourth and fifth rounds aimed to confirm the consensus and recommendations from the previous rounds. To validate the importance of the proposed indicators among a broad group of maternal health stakeholders, round 4 was a request for public comment. This call for public comments was posted online on the Maternal Health Task Force website (MHTF.org) for a period of two weeks, and was disseminated by email to multiple networks. The request included an online survey in which participants were asked to confirm, on a 4-point scale (“extremely important,” “moderately important,” “not at all important,” “don’t know/no opinion”), the importance of each of the indicators in the draft set of Phase II core indicators. Public comment participants were also given the opportunity to submit additional indicators for consideration, provided that the indicator they submitted was not one that had been evaluated and eliminated during previous rounds, and that the submission included full information and meta-data for that indicator. Nine additional indicators were submitted via the request for public comment; after review by the steering committee, three of those indicators were eliminated due to incomplete information or duplication with indicators already included in the draft set.

The fifth and final round in this indicator development process was an in-person, expert meeting to review and confirm consensus on the draft set of indicators. The meeting, hosted by the Maternal Health Task Force, included 48 participants, representing country, technical, and policy-making perspectives; many of the attendees were also involved with the prior rounds of review. Prior to the consultation, duplicates were removed from the draft indicator set, minor modifications were highlighted, and a set of outstanding questions for resolution by consensus was drafted.

Ultimately, 150 experts from more than 78 organizations participated in this second phase of the process to develop a comprehensive monitoring framework for EPMM (Additional file 2). Guided by the 11 key themes outlined in the EPMM Strategies report, experts considered a total of 118 indicators related to the social, political, and economic determinants of maternal health and survival.

## Results

At the end of this process, consensus was obtained on 27 indicators and a set of six stratifiers. The stratifiers were selected to enable the tracking of equity and transparency. After removing duplicates, a total of 25 indicators and six stratifiers comprise the final set of core indicators for Phase II (Table 4). The definitions, recommended disaggregators, and data sources for the final set of indicators can be found in the indicator meta-data (Additional file 3). Unlike Phase I of the process to develop a monitoring framework for ending preventable maternal mortality, which included input from 45 experts and resulted in 12 core indicators, the Phase II process was much larger in scope; it ultimately included input from three times as many experts and resulted in double the number of indicators.

**Table 4:** EPMM Phase II Core Indicators

Indicator	
Presence of laws and regulations that guarantee women aged 15-49 access to sexual and reproductive health care, information, and education	
Gender Parity Index (GPI)	
Whether or not legal frameworks are in place to promote, enforce, and monitor equality and non-discrimination on the basis of sex	
Presence of protocols/policies on combined care of mother and baby, immediate breastfeeding, and observations of care	
Maternity protection in accordance with ILO Convention 183	
International Code of Marketing of Breastmilk Substitutes	
Costed implementation plan for maternal, newborn, and child health	
Midwives are authorized to deliver basic emergency obstetric and newborn care	
Legal status of abortion	
Proportion of women aged 15-49 who make their own informed decisions regarding sexual relations, contraceptive use, and reproductive health care	
Geographic distribution of facilities that provide basic and comprehensive emergency obstetric care (EmOC)	
Presence of a national set of indicators with targets and annual report to inform annual health sector reviews and other planning cycles	
Maternal death review coverage	
Percentage of total health expenditure spent on reproductive, maternal, newborn, and child health	
Out-of-pocket expenditure as a percentage of total expenditure on health	
Annual reviews are conducted of health spending from all financial sources, including spending on RMNCH, as part of broader health sector reviews	
Health worker density and distribution (per 1000 population)	
Coverage of essential health services	
If fees exist for health services in the public sector, are women of reproductive age (15-49) exempt from user fees for [MH-related health] services	
Demand for family planning satisfied through modern methods of contraception	
Availability of functional emergency obstetric care (EmOC) facilities	
Density of midwives, by district (by births)	
Percentage of facilities that demonstrate readiness to deliver specific services: family planning, antenatal care, basic emergency obstetric care, and newborn care	
Civil registration coverage of cause of death (percentage)	
Presence of a national policy/strategy to ensure engagement of civil society organization representatives in periodic review of national programs for maternal, newborn, child, and adolescent health (MNCAH)	
Standard Equity Stratifiers	
Wealth	
Area of residence: urban/rural	
Area of residence: geographic region	
Level of education: women’s education level	
Age	
Transparency Stratifier	
Available in the public domain	

The Phase II indicators are well harmonized with the Indicator and Monitoring Framework for the Global Strategy, SDGs 3 and 5, and Countdown to 2015. Of the 25 indicators, 5 overlap with the Global Strategy, 14 overlap with the SDGs, and 11 overlap with Countdown to 2015. (Table 5) Such harmonization is key to advancing EPMM’s objective of supporting achievement of the SDGs and the Global Strategy and complementing other global monitoring efforts.

**Table 5:** EPMM Phase II Core Indicators Harmonized with Other Monitoring Efforts

**Indicator**	SDGs	GS	CD
Presence of laws and regulations that guarantee women aged 15-49 access to sexual and reproductive health care, information, and education	✓	✓	
Gender Parity Index (GPI)		✓	
Whether or not legal frameworks are in place to promote, enforce, and monitor equality and non-discrimination on the basis of sex	✓	✓	
Presence of protocols/policies on combined care of mother and baby, immediate breastfeeding, and observations of care			
Maternity protection in accordance with ILO Convention 183			✓
International Code of Marketing of Breastmilk Substitutes			✓
Costed implementation plan for maternal, newborn, and child health			✓
Midwives are authorized to deliver basic emergency obstetric and newborn care			✓
Legal status of abortion	✓		✓
Proportion of women aged 15-49 who make their own informed decisions regarding sexual relations, contraceptive use, and reproductive health care	✓	✓	
Geographic distribution of facilities that provide basic and comprehensive emergency obstetric care (EmOC)			
Presence of a national set of indicators with targets and annual report to inform annual health sector reviews and other planning cycles	✓		
Maternal death review coverage	✓		
Percentage of total health expenditure spent on reproductive, maternal, newborn, and child health	✓		
Out-of-pocket expenditure as a percentage of total expenditure on health	✓		✓
Annual reviews are conducted of health spending from all financial sources, including spending on RMNCH, as part of broader health sector reviews			
Health worker density and distribution (per 1000 population)	✓	✓	
Coverage of essential health services			
If fees exist for health services in the public sector, are women of reproductive age (15-49) exempt from user fees for [MH-related health] services			
Demand for family planning satisfied through modern methods of contraception			
Availability of functional emergency obstetric care (EmOC) facilities			
Density of midwives, by district (by births)			
Percentage of facilities that demonstrate readiness to deliver specific services: family planning, antenatal care, basic emergency obstetric care, and newborn care			
Civil registration coverage of cause of death (percentage)	✓		
Presence of a national policy/strategy to ensure engagement of civil society organization representatives in periodic review of national programs for maternal, newborn, child, and adolescent health (MNCAH)	✓		
**Stratifiers: Equity**			
Wealth			✓
Area of residence: urban/rural			✓
Area of residence: geographic region			✓
Level of education: women’s education level			✓
Age			✓
**Stratifier: Transparency**			
Available in the public domain			

*SDGs* Sustainable Development Goals

*GS* Global Strategy for Women’s, Children’s and Adolescents’ Health (2016-2030)

*CD* Countdown to 2015

A small number of outstanding issues remained with regard to specific indicators, and were resolved through a facilitated, semi-structured debate among participants at the final expert meeting. The issues that guided this debate and their resolutions are listed below.

1. Throughout the webinars and surveys, participants recommended “minor modifications” to some indicators. However, some of these recommended modifications had potentially significant implications for definition, data collection and measurement. The group discussed whether modification of any kind should warrant removal to the additional set of indicators for further development. The group consensus was to keep these indicators on the core list in their original form (without modification) and to add the indicators along with the recommended modifications to a secondary list of additional indicators for further development.
2. An indicator tracking the presence and reporting of data as described by ICD-PM^2^ was aspirational, with no previous publication or use [22]. The group agreed that this indicator should be removed to the list of additional indicators for further development.
3. Six new indicators that were submitted through the request for public comment were considered. These indicators did not go through the same rigorous iterative process of expert evaluation according to selection criteria and prioritization via the modified Delphi method. The expert group discussed and agreed on next steps for each indicator, eliminating four due to duplication, incomplete information or failure to meet selection criteria; advancing one to the list of additional indicators for further development; and adapting one to develop a stratifier focused on transparency.

An important secondary outcome of this iterative process was the identification and prioritization of the list of additional indicators that all participants in this process agreed are relevant, important, and useful for tracking progress toward EPMM strategic priorities, but that require further development and research before they can be recommended for global monitoring and national reporting. A total of 30 indicators fell into this category. This process and the indicators that emerged from it will be described in a separate publication.

## Discussion

The process and outcomes described above are a timely and important contribution to global maternal health monitoring. They address the lack of measures for the social, political, and economic determinants of maternal health and survival, complement other maternal health monitoring efforts at the policy, system, and facility level, and provide a framework to support countries as they endeavor to achieve the maternal health target set by the SDGs.

Maternal health and survival are situated within the broader context of a woman’s full life course, including but not limited to adolescence and sexual and reproductive health. This continuum cannot be addressed in isolation from the social and political dynamics and structural inequalities that influence the systems in which women not only live, but also seek and receive healthcare [23, 24]. The SDGs and the Global Strategy place emphasis on poverty reduction, gender equality, universal health coverage, and a human rights approach to health, exemplified by attention to the fundamental human rights principles of equity and non-discrimination, transparency, participation, and accountability. Nevertheless, several commentaries highlight the lack of sufficient global-level work on the development of measures for the more distal determinants of health as we enter the SDG period [25, 26]. Indeed, most global and national monitoring frameworks focus heavily on indicators that track health status and service coverage. For example, the WHO Global Reference List of 100 Core Health Indicators largely lacks indicators to track distal determinants of health outcomes beyond measures of health system status—such as enabling laws and policies, and social determinants like education, gender, and socio-economic barriers that impact on health status [27].

We acknowledge the critical importance of ongoing work to determine the best measures to drive facility, community, national, and regional progress. But it is clear that coverage and quality of essential clinical interventions at the bedside (e.g. immediate administration of uterotonics after birth), and the attendant improvement in health outcomes at the client level (e.g., effective prevention of postpartum hemorrhage), are highly dependent on upstream factors such as adequate health workforce (e.g. density of midwives), enabling policies (e.g. midwives are authorized to deliver basic emergency obstetrics and newborn care) and facility readiness (e.g. a reliable supply chain for essential commodities). These factors, in turn, are affected by structural social, political, and economic factors, such as the status of women in societies, measurement capacity and data quality for effective surveillance and response, and adequate allocation of resources to maternal health.

The burden on individual providers of collecting data has been well documented [28, 29], as has the lack of use of data collected at such great cost [30–32], which breaks the feedback mechanism whereby monitoring and review can result in improved provision of interventions. Global level indicators to address social determinants of health may seem distal, too, from the day to day process of managing clinical care. Because the indicators identified here were designed to tackle the social and distal determinants of care, and aim to address determinants of health that lie upstream from the most immediate factors which influence a woman’s health outcome, they may seem beyond the scope of influence of the individual provider even though typically, that provider lives and acts in the same environment and is affected by the same cultural norms. The results of tracking progress on social determinants may not appear, at first blush, as immediate as counting the number of women treated for PPH, but over time, increases in girls’ educational attainment may well prove greatly significant in ending preventable deaths [33, 34].

It is clear that global policies and strategies must be grounded in the realities and challenges of care in settings where women are dying. Real change, however, must be systemic and will only come when the concept that no woman should die in pregnancy or childbirth is engrained throughout every culture and society as a fundamental right and an indisputable truth. Therefore, the tripartite components of accountability adopted by the Independent Accountability Panel—monitor, review, and act—must be applied at every level from critical distal determinants of maternal health and survival to those at the bedside level in order to ensure high-quality, high-performing health systems that are able to ensure the highest attainable level of health for all.

Underscoring the need for more work in this area, in March 2017 WHO established a *Global RMNCAH Policy Reference Group* (PRG) charged with advising WHO on which policies to monitor under the umbrella of the Global Strategy. In this context, the work described above to identify relevant, useful, valid and feasible maternal health-specific indicators for less-developed global monitoring areas such as health financing, laws, and policies was especially timely and important.

There are a number of global efforts to improve maternal and newborn health monitoring at the policy, system, and facility levels and the process to develop the Phase II core indicators complemented these efforts well. At the policy and systems level, for example, representatives from the High-Level Working Group on Health and Human Rights, the Commission on Social Determinants of Health, the Global Financing Facility, the Countdown to 2030 Working Group on Drivers, and the WHO Health Policy Reference Group were all included in several rounds of the Phase II process, participating in webinars, surveys, and the expert meeting, consulting on relevant themes, and receiving information on the process’s outcomes through direct outreach. This helped to ensure that the Phase II process was well-coordinated with the aforementioned groups’ efforts to implement the Office of the High Commissioner of Human Rights (OHCHR)’s “Technical guidance on the application of a human rights-based approach to the implementation of policies and programmes to reduce preventable maternal mortality” [35]. In addition, coordination with the WHO Quality of Care Network, Every Newborn Action Plan (ENAP), Improving Coverage Measurement, and the Countdown to 2030 ensured that the Phase II process also complemented efforts aimed at driving improvements at the facility level [36].

The outcomes of the Phase II process also complement a number of other maternal and newborn monitoring efforts, not least of which are the SDGs and Global Strategy. Upon completion, the set of EPMM Phase II indicators was delivered to the Mother Newborn Information for Tracking Outcomes and Results (MONITOR) expert review group^3^, which was recently formed by the WHO and tasked with advising the WHO on maternal and newborn health monitoring, mapping the full complement of available metrics for maternal newborn health monitoring, and providing technical guidance for the incorporation of those indicators into routine use at country level. The Phase II set of indicators has also been used in the development of the Countdown to 2030’s indicator lists. Future steps for the Phase II set include targeted testing and validation of the indicators developed during this process and support for their incorporation into global and national monitoring frameworks and data systems for routine use.

There are numerous risks to progress for maternal health in the current geopolitical context. The global framework put forward in the SDGs is much broader than that of the MDGs, with many more goals and targets; there is a risk that the unfinished maternal health agenda could fall through the cracks in the face of many more competing priorities. Furthermore, there is also a risk of sliding backwards on women’s sexual and reproductive health and rights, which would have significant repercussions for maternal health and survival [37]. Now more than ever, attention is needed to ensure that maternal health and survival remain high on the global development agenda and tools and resources are readily available to ensure effective, strategic action to achieve the goal of ending preventable maternal deaths within a generation.

Fortunately, though collecting the indicator data may be challenging, the EPMM Phase II indicators have multiple implications for practical application. It is hoped that they will be useful for national planning, reporting, and monitoring, as well as cross-ministerial work, “health in all” policies, and other best practices regarding strategic planning and decision-making. They can provide a concrete monitoring framework for priority recommendations aimed at achieving strengthened health systems. These indicators acknowledge that health service quality is shaped at all levels of the health system. Especially for formidable and complex goals such as ensuring universal health coverage of comprehensive sexual, reproductive, maternal and newborn care, it is hoped that the indicators we propose can provide a means of implementation for achieving and tracking progress toward their progressive realization.

To further foster action at all levels of the health system, the indicators may also be applied in the context of social accountability and advocacy, an approach supported by the recommendations of the International Initiative on Maternal Mortality and Human Rights, which calls for a rights-based approach to maternal mortality reduction [37, 38]. Finally, they are intended to be useful for global monitoring and reporting and thus to support achievement of the SDGs and accountability for the full realization of all three pillars of the Global Strategy in the specific context of maternal health and survival.

Keeping in mind that ending preventable maternal mortality is a country-driven endeavor, stakeholder recommendations on the uses and target audiences for the final set of EPMM Phase II indicators were compiled. Participants in the expert meeting in particular proposed several suggestions to improve the presentation and user-friendliness of Phase II core indicator list. Suggestions included:

1. A comprehensive list of EPMM indicators that includes the indicators from both Phases I and II;
2. Lists that display the indicators by harmonization with other monitoring frameworks, key theme, and maternal health topic area; and
3. Operational guidance to facilitate the prioritization, selection, and use of EPMM indicators at the country level based on context-specific needs

The first two suggestions have been addressed. The comprehensive list of Phase I and II indicators can be found in Table 6. Lists displaying the Phase II indicators by key theme, harmonization with other monitoring frameworks, and maternal health topic area can be found in Additional file 1, Table 5, and Additional file 4, respectively. Mechanisms to address country requests for operational guidance to facilitate context-specific use of the indicators are under development by the EPMM Working Group.

**Table 6:** EPMM Phase I and Phase II Core Indicators

Phase I Indicators	Phase II Indicators
**Coverage**	
Four or more antenatal visits	Proportion of women ages 15-49 who make their own informed decisions regarding sexual relations, contraceptive use, and reproductive health care
Skilled attendant at birth	Maternal death review coverage
Institutional delivery	Coverage of essential health services
Maternal death registration	Demand for family planning satisfied through modern methods of contraception
Early postnatal/postpartum care for woman and baby (within 2 days of birth)	Civil registration coverage of cause of death (percentage)
Met need for family planning	
Uterotonic immediately after birth	
Caesarean rate	
**Health Systems Strengthening & Finance**	
Availability of functional emergency obstetric care facilities	Availability of functional emergency obstetric care facilities
	Geographic distribution of facilities that provide basic and comprehensive emergency obstetric care
	Health worker density and distribution (per 1000 population)
	Density of midwives, by district (by births)
	Percentage of facilities that demonstrate readiness to deliver specific services: family planning, antenatal care, basic emergency obstetric care, and newborn care
	Percentage of total health expenditure on reproductive, maternal, newborn, and child health
	Out-of-pocket expenditure as a percentage of total expenditure on health
**Impact**	
Maternal mortality ratio	
Maternal cause of death (direct/indirect) based on ICD-MM	
Adolescent birth rate	
**Policy**	
	Presence of laws and regulations that guarantee women ages 15-49 access to sexual and reproductive health care, information, and education
	Gender Parity Index
	Whether or not legal frameworks are in place to promote, enforce, and monitor equality and non-discrimination on the basis of sex
	Presence of protocols/policies on the combined care of mother and baby, immediate breastfeeding, and observations of care
	Maternity protection in accordance with ILO Convention 183
	International Code of Marketing of Breastmilk Substitutes
	Costed implementation plan for maternal, newborn, and child health
	Midwives authorized to deliver basic emergency obstetric and newborn care
	Legal status of abortion
	Presence of a national set of indicators with targets and annual report to inform annual health sector reviews and other planning cycles
	If fees exist for health services in the public sector, women of reproductive age (15-49) are exempt from user fees for maternal health-related health services
	Annual reviews are conducted of health spending from all financial sources, including spending on RMNCH, as part of broader health sector reviews
	Presence of a national policy/strategy to ensure engagement of civil society organization representatives in periodic review of national programs for maternal, newborn, child, and adolescent health
**Equity and Transparency (Stratifiers)**	
	Wealth
	Area of residence: urban/rural
	Area of residence: geographic region
	Level of education: women’s education level
	Age
	Available in the public domain

This process included both strengths and limitations. One strength of this project was the use of a rigorous, systematic, and iterative process based on sound methodology. Another strength was the broad participation from maternal health stakeholders worldwide, which was achieved via active outreach to numerous constituencies and experts in sexual, reproductive maternal and newborn health, human rights, health policy, workforce planning, measure development, clinical quality improvement, health economics and financing, epidemiology, demography and health statistics and other relevant domains. Participants were from government agencies and non-governmental organizations, national Ministries of Health, bilateral and donor organizations, academic and research institutions, policy think tanks, clinical care facilities and program administration, among others.

There were also some limitations to this process. First, the number of participants at each stage was limited despite concerted efforts to be inclusive and representative. Second, many indicators recommended for inclusion in the final set have not yet been validated and tested at the national level. As noted above, however, work is currently being planned to test and validate these indicators. Finally, many indicators considered by the experts involved in this process were deemed important, relevant, and useful for tracking progress in key thematic areas but still in need further development before being recommended for monitoring at global and national levels. Those indicators could not be included in the core set of indicators. Nevertheless, this set of additional indicators for further development represents an important agenda for future research in the area of measure development for maternal newborn health monitoring, and provides a strong basis and rationale for the need for further work in this area.

Finally, a point of frequent discussion among participants in this process was that the mere presence of a policy does not indicate its effective implementation or impact. The policy indicators recommended here provide a point of entry for monitoring in these areas. Monitoring the presence of policies that aim to improve maternal health and survival establishes a basis for advocacy and holds policy makers to account for the effective implementation of said policies, as well as, when necessary, effective redress in the event of non-compliance. Consistent with the human rights concept of progressive realization, therefore, we recognize that indicators that capture the existence of policies addressing the social determinants of maternal health and survival, while necessary, may not be sufficient and look forward to further progress toward ensuring measures of effective implementation, and eventually, measures that track the impact of such policies [26]. We look forward to the work of the recently established WHO Policy Reference Group in this area. At the same time, we hope that providing even an imperfect entry point for monitoring critical distal determinants of maternal health and survival will represent a useful contribution toward creating the enabling environment for functional health systems that are able to deliver high-quality care to all women and end preventable maternal deaths. Greater attention is being given to the roles that poorly functioning health systems and unaddressed upstream factors play in creating barriers to the provision of critical lifesaving interventions by frontline workers [3]. The role of the health system has also been described and specifically called out in the process of refining the definition of skilled attendance at birth [39]. The resulting moral distress and burnout they face [40, 41] thus highlights yet another way in which these distal determinants significantly influence provision and experiences of care at the facility level where maternal and perinatal deaths and disabilities occur and the rights of women are violated.

## Conclusion

Ending preventable maternal mortality and correcting unacceptable levels of disparity are essential to achieving SDG 3, which focuses on global health for all. Considering the critical role women play in families, economies, and societies, and in the development of future generations and communities, their needs cannot be ignored. Now is a time of both opportunity and threat for the global maternal health agenda. There is a very real risk that the focus needed to improve maternal health and survival will be lost in the broad new SDG framework and the unfinished agenda for maternal health will not be completed. At the same time, there is research suggesting that we could end preventable maternal deaths within a generation and achieve “a grand convergence” by eliminating wide disparities in current maternal mortality and reducing the highest levels of maternal deaths worldwide to rates now observed in the best-performing middle-income countries [42].

In concluding our paper on Phase I of the process to develop a comprehensive monitoring framework for EPMM we cited the adage, “What gets measured gets done” [11]. The broad focus of the EPMM Strategies addresses not only the essential clinical interventions needed to save lives but also the equally important political, social, economic and health system determinants of maternal health and survival. These factors are essential to creating the enabling environment and high-performing health systems needed to ensure high-quality clinical care at the point of service for every woman, her fetus and newborn. Without a monitoring framework and robust measures for tracking progress in these more distal areas for improvement, their importance remains largely rhetorical. This paper complements Phase I by rounding out the set of core maternal health indicators focused on coverage and impact of key interventions closely linked to the more proximal causes of death with a set of maternal health policy and health system indicators focused on the more distal, yet still critical social, political, and economic determinants of maternal health and survival highlighted in the EPMM Strategies.

The indicators identified through the process described above provide a concrete tool to support the implementation of monitoring progress towards each of the 11 key themes outlined EPMM Strategies report. It is hoped that national and global decision makers and program planners will find them to be useful tools for accelerating progress toward eliminating the disparities driven by social determinants, structural inequalities, and system deficiencies that contribute to preventable maternal deaths around the world. Together, the EPMM Strategies and its accompanying monitoring framework—developed through the consultative process described above—support achievement of the SDGs and Global Strategy within the specific domain of maternal health.

## Additional files

Additional file 1: EPMM Phase II Indicators by Key Theme. (DOCX 1222 kb)

Additional file 2: List of Participating Organizations. (DOCX 27 kb)

Additional file 3: EPMM Phase II Indicators and Meta-Data. (PDF 107 kb)

Additional file 4: Phase II Indicators by Maternal Health Topic Area. (DOCX 33 kb)

## Abbreviations

ENAP: Every Newborn Action Plan
EPMM: Ending Preventable Maternal Mortality
ICD-PM: International Classification of Diseases – Perinatal Mortality
MDG: Millennium Development Goal
MHTF: Maternal Health Task Force
MMR: Maternal Mortality Ratio
MONITOR: Mother Newborn Information for Tracking Outcomes and Results
OHCHR: Office of the High Commissioner for Human Rights
SDG: Sustainable Development Goal
UNFPA: United Nations Population Fund
USAID: U.S. Agency for International Development
WHO: World Health Organization

## Footnotes

1 A coalition made up of the World Health Organization and partner organizations which include the FCI Program of Management Sciences for Health, Jhpiego, Maternal and Child Survival Program, Maternal Health Task Force, UNFPA, UNICEF, USAID, and White Ribbon Alliance.

2 WHO describes ICD-PM as “the WHO application of ICD-10 deaths during the perinatal period… it is intended to facilitate the consistent collection, analysis, and interpretation of information on perinatal deaths.”

3 The Terms of Reference for the newly formed MONITOR expert group can be found here: http://www.who.int/maternal_child_adolescent/news_events/news/tor-monitor-expert-advisory-group.pdf?ua=1.
