# Supplementary material for: Ending preventable maternal mortality: phase II of a multi-step process to develop a monitoring framework, 2016–2030
Source: BMC Pregnancy Childbirth. 2018 Jun 25;18:258. doi: 10.1186/s12884-018-1763-8 (PMC6019318; doi:10.1186/s12884-018-1763-8)
Supplement: Supplementary file 1 — EPMM Phase II Indicators by Key Theme. (DOCX 1222 kb) [file 12884_2018_1763_MOESM1_ESM.docx]

Additional File 1. EPMM Phase II Indicators by Key Theme


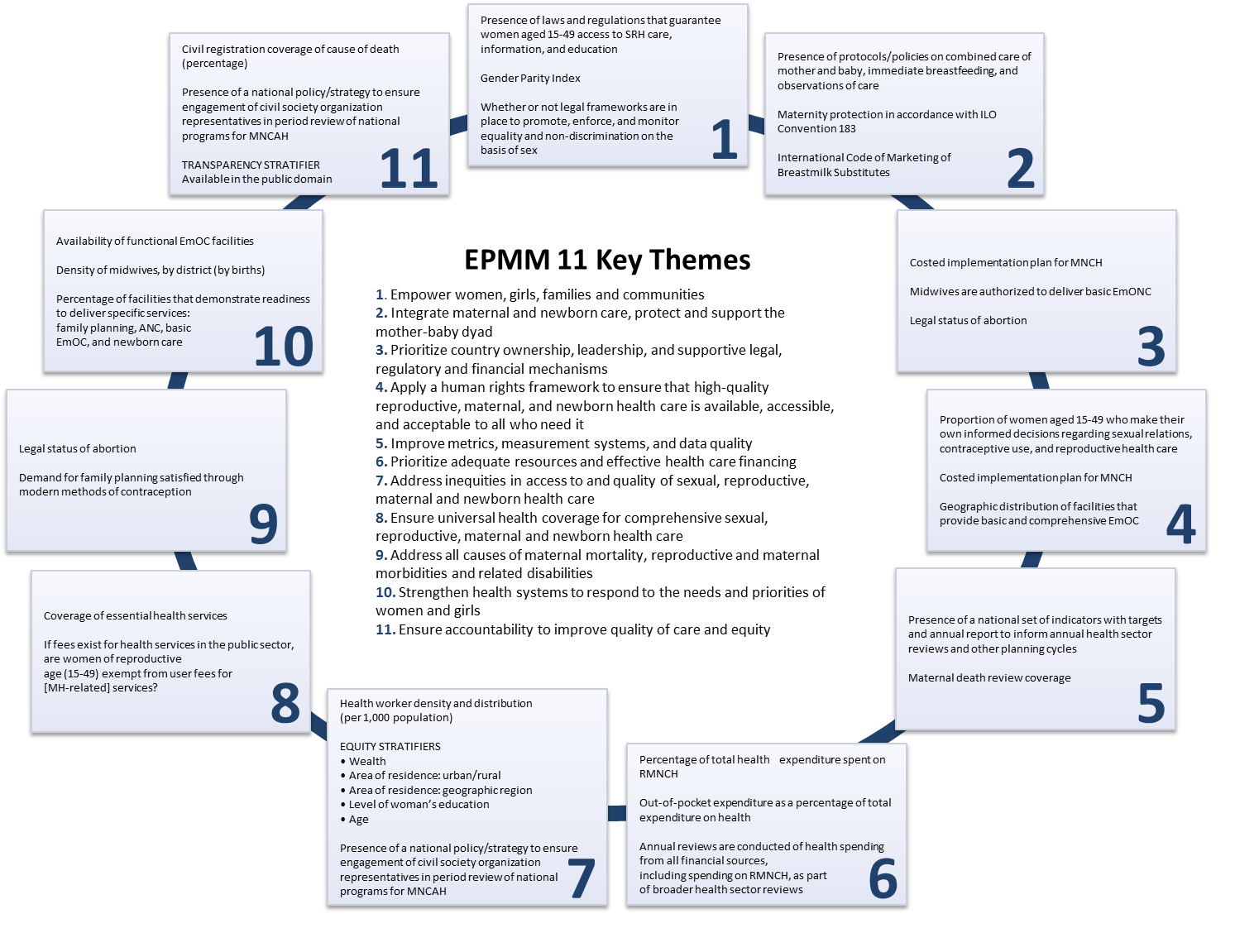


| **Theme 1: Empower women, girls, families and communities** |
| --- |

**Priority Recommendations**

Gender Equality

- There is equal access to resources
- There is equal access to education and information (including comprehensive sexuality education)
- There are traditional or social media campaigns to help change social norms in families and communities
- There are programs for engaging men and boys as supporters and change-agents

Violence and Discrimination

- There are focused efforts to eliminate gender-based violence and discrimination, including disrespect and abuse of women using health care services

Participation and Representation

- Governments address structural, historical and social determinants of health and gender discrimination, including economic inequality and workplace discrimination to ensure substantive equality
- Women and communities have the ability to influence the quality of services through participatory mechanisms and social accountability

Sexual and Reproductive Health and Rights

- Women have not only have the power of decision making for SRMNH but also the availability of options that allows them to exercise their choices
- Women and girls have rights to delay marriage and childbearing

**Indicator Identification Process**

**
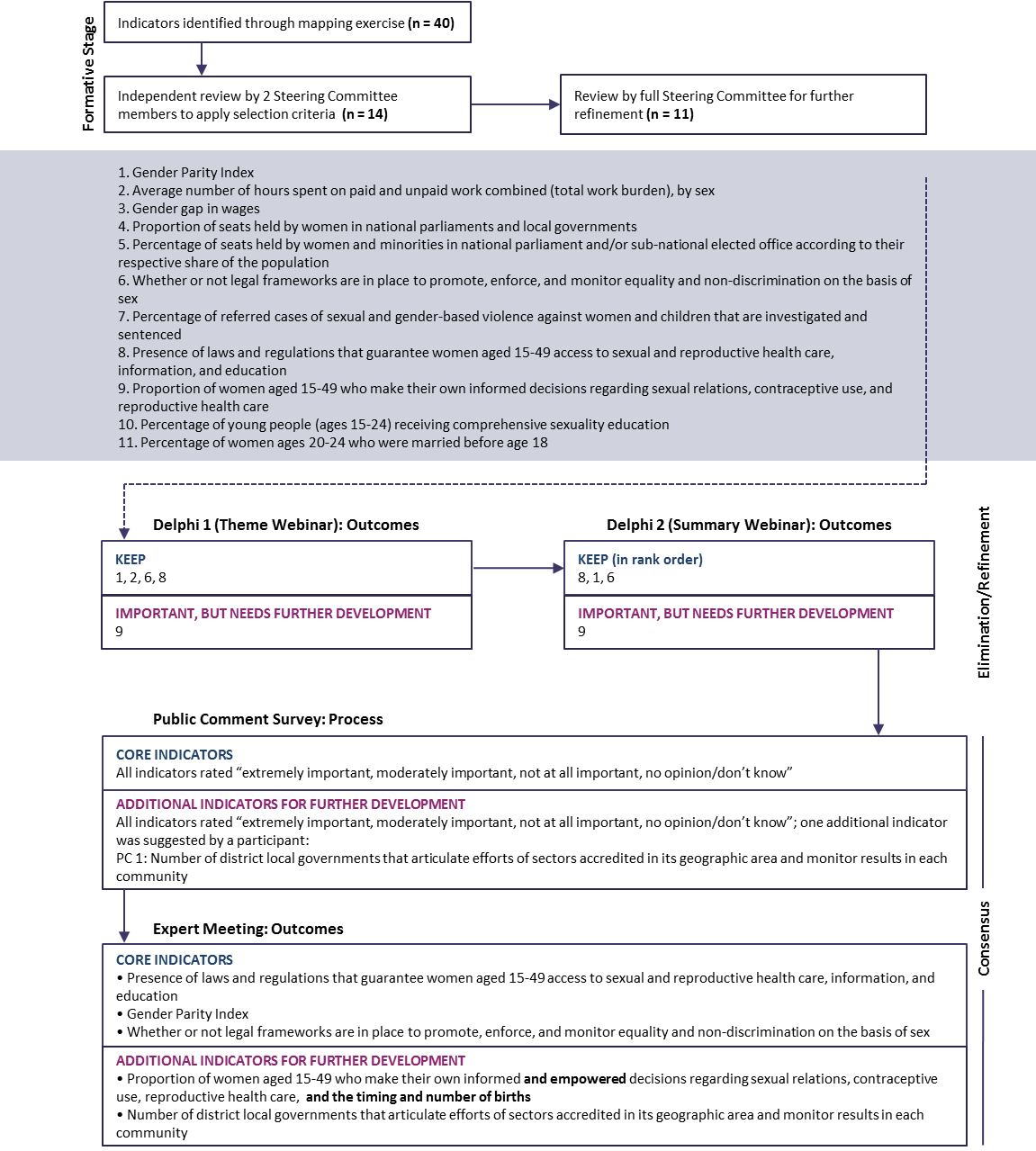
**

| **Theme 2: Integrate maternal and newborn care, and protect and support the mother-baby relationship** |
| --- |

**Priority Recommendations**

Integrated Maternal and Newborn Health Services

- There is integration of strategies for both mother and baby
- There is integration of service delivery (including PMTCT) for both mother and baby
- MNH services are delivered together whenever this can be done without compromising quality of care for either: necessary care is provided without separation of the baby from its mother

Linked Registration of Births and Deaths

- There is linkage of vital registration data collected for mothers and their newborn

**Indicator Identification Process**


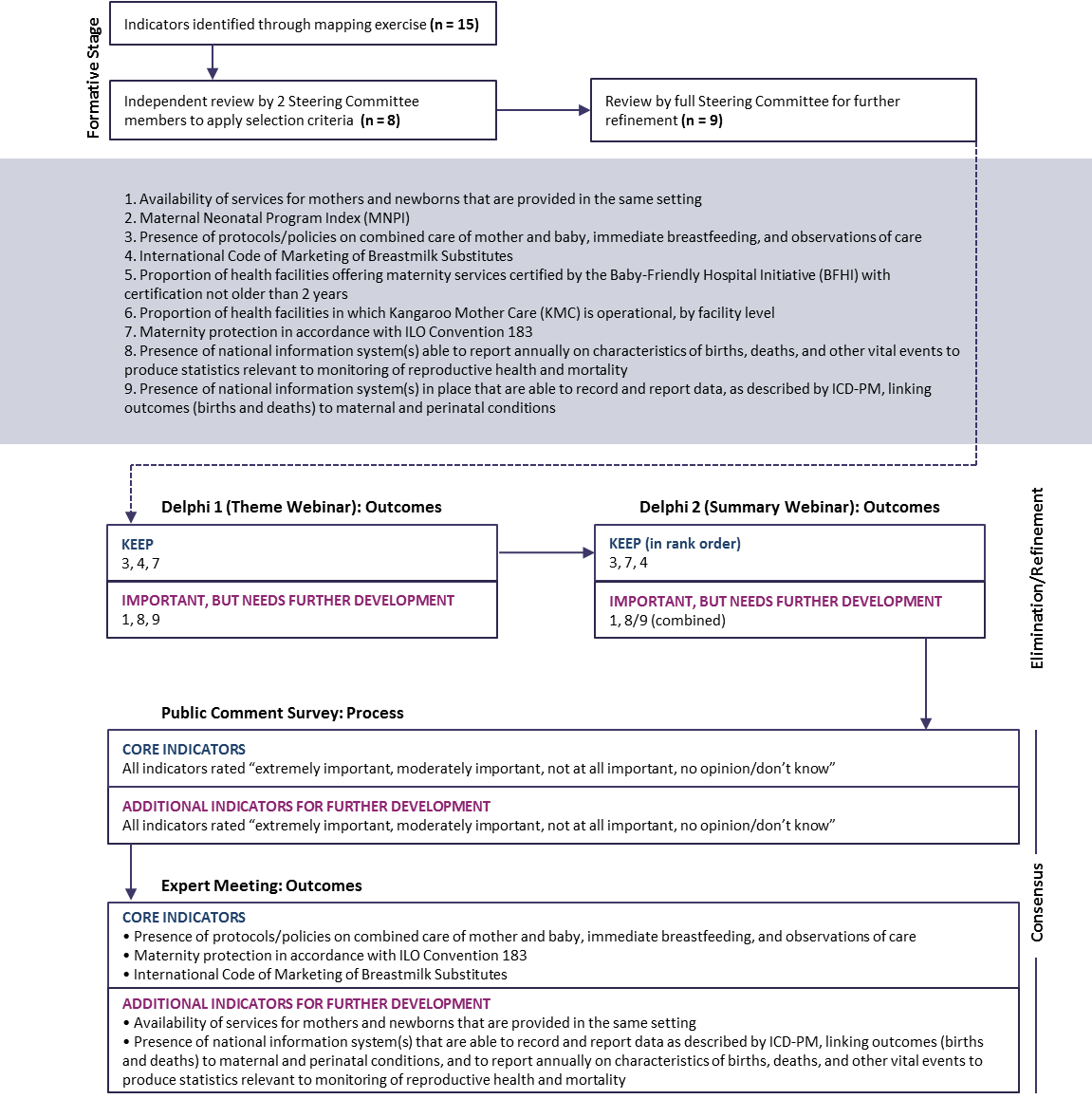


| **Theme 3: Prioritize country ownership, leadership, and supportive legal, regulatory, and financial mechanisms** |
| --- |

**Priority Recommendations**

Country Ownership and Leadership

- There is evidence of strong country leadership: policies and financial commitments by country leaders
- There is evidence of strong leadership to champion global and country MMR targets
- There is good governance and effective stewardship of the full array of political tools, social capital and financial resources available to support and enable a high-performing health system
- There is community input and participation via social accountability mechanisms that encourage women and communities to participate in the system

Supportive Legal Frameworks

- There are mechanisms for legal redress for those harmed, abused or abandoned in the course of seeking care
- There are laws and policies that uphold human rights in the context of maternal health care
- There are laws that guarantee access to comprehensive MH care and provide for universal health coverage (UHC)
- There are supportive employment laws and frameworks for legal licensure of the maternity care workforce within the jurisdictions where they are needed
- There are laws that address gender discrimination and empower women and girls

Supportive Financial Frameworks

- There is transparent, publicly available information on maternal health budgets and policies
- There is donor harmonization and efforts by donors to ensure that funding does not impose structural barriers to the achievement of important outcomes not readily measured within short funding cycles or along vertical technical and program lines
- There are financial mechanisms aimed at achieving UHC include conditional cash transfers, voucher programs, various forms of insurance and performance-based incentives

Supportive Regulatory Frameworks

- There is collection of vital statistics and improved data on causes of maternal and newborn deaths and stillbirths through MDSR
- There are regulatory mechanisms to enable effective human resources management of the necessary workforce, such as regulation of midwives, nurses and doctors, task sharing

**Indicator Identification Process**

**
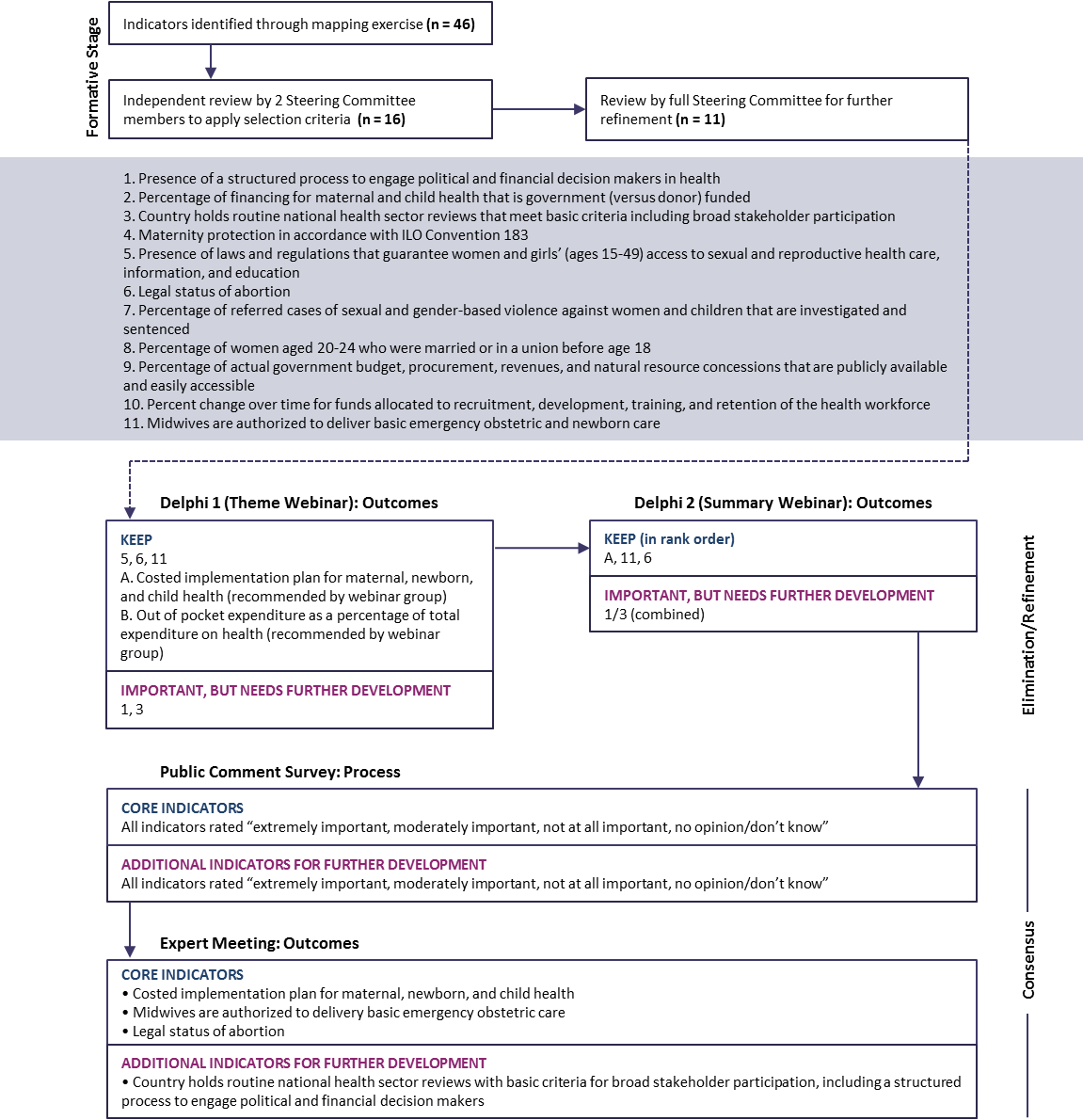
**

| **Theme 4: Apply a human rights framework to ensure that high-quality reproductive, maternal, and newborn health care is available, accessible, and acceptable to all who need it** |
| --- |

**Priority Recommendations**

Maternal Health and Survival as a Reflection of the Right to Health

- States include the prevention of maternal mortality and the provision of maternal health services within their obligations to fulfill the right to health
- States ensure maternal health care for women—which includes pre-natal and post-natal care—as a core obligation under the right to health
- States realize the Office of the UN High Commissioner for Human Rights (OHCHR) technical guidance on implementing a rights-based approach to maternal health programming and policy

Right to Maternal Healthcare as a Reflection of Gender Equality and Non-Discrimination

- A rights-based approach includes empowerment, participation, non-discrimination, transparency, sustainability, accountability and international assistance as fundamental principles
- States ensure equality of health results for women—including lowering of maternal mortality rate—as part of realization of women’s rights
- Where resources are limited, states prioritize interventions that will help guarantee maternal health and in particular emergency obstetric care

Accessibility, Acceptability, Availability, and Quality of Care (AAAQ)

- Governments take steps to improve child and maternal health, sexual and reproductive health services, including access to family planning, pre- and post-natal care, emergency obstetric services and access to information, as well as to resources necessary to act on that information
- Plans go beyond focusing solely on prevention of worst outcomes for women at highest risk towards supporting and encouraging optimal outcomes for all women

**Indicator Identification Process**


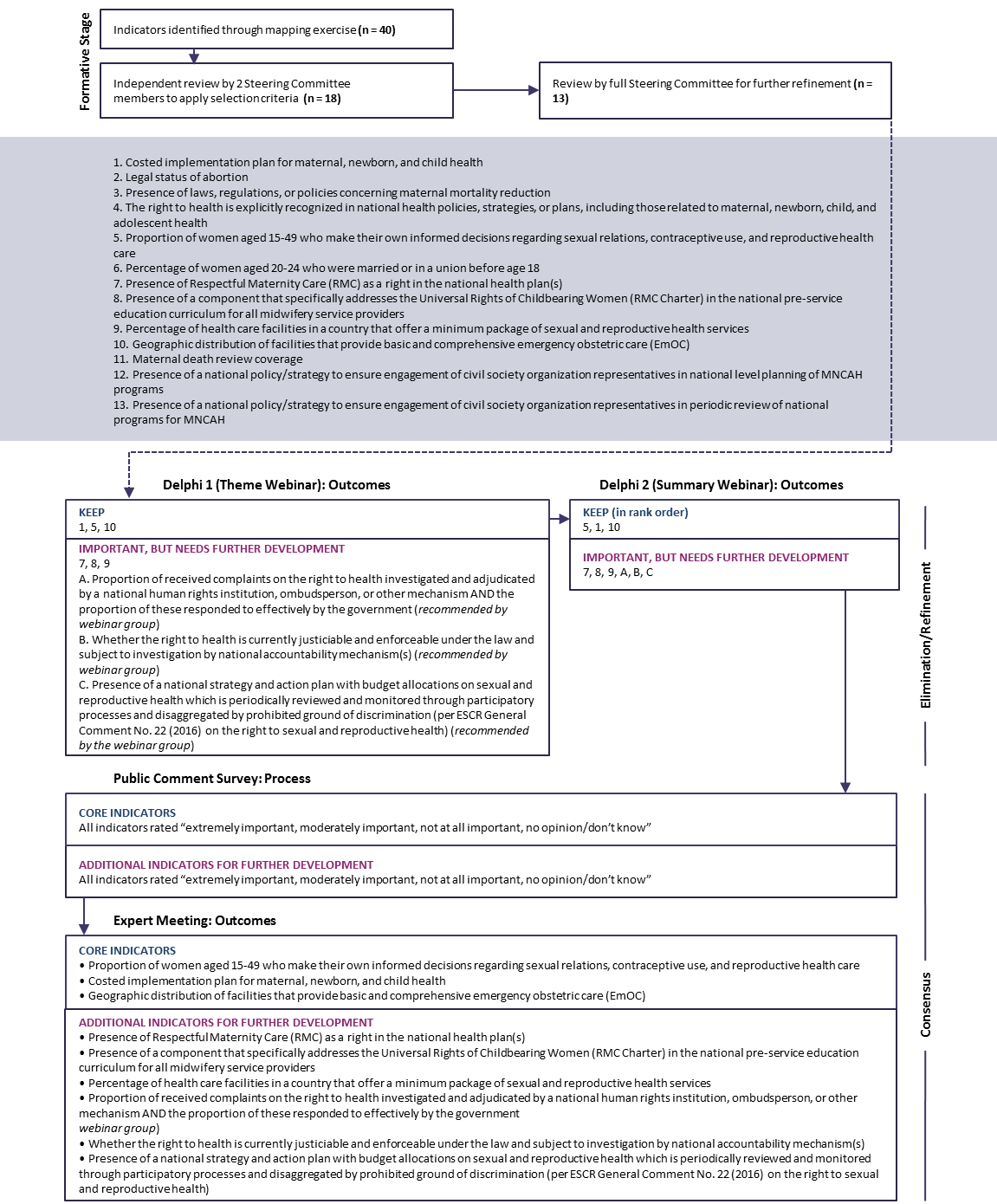


| **Theme 5: Improve metrics, measurement systems and data quality** |
| --- |

**Priority Recommendations**

Counting Deaths

- All maternal and perinatal deaths are counted through effective national registration and vital statistics systems in every country
- National registries link the data of mothers and their newborns
- All countries have a complete civil registration system with accurate attribution of the cause of death
- Countries have established MDSR and similar perinatal death surveillance mechanism

Data Quality

- Countries use standardized instruments and indicators to track maternal mortality using definitions that are consistent with the current International Classification of Diseases manual (ICD-MM)
- Countries use standardized data sources, indicators and intervals for data collection to allow for better global comparisons
- Countries use technologies for data collection (e.g. mapping, mobile phones) with shown effectiveness for speeding up data collection to allow effective, real-time use

Data-driven Program and Service Improvement

- Countries have established confidential inquiries and collection of quality of care data on near misses and severe morbidities
- Countries use improved maternal mortality metrics and measurement systems for the purpose of accountability to track equity
- Countries use improved maternal mortality metrics and measurement systems to ensure program effectiveness

**Indicator Identification Process**

**
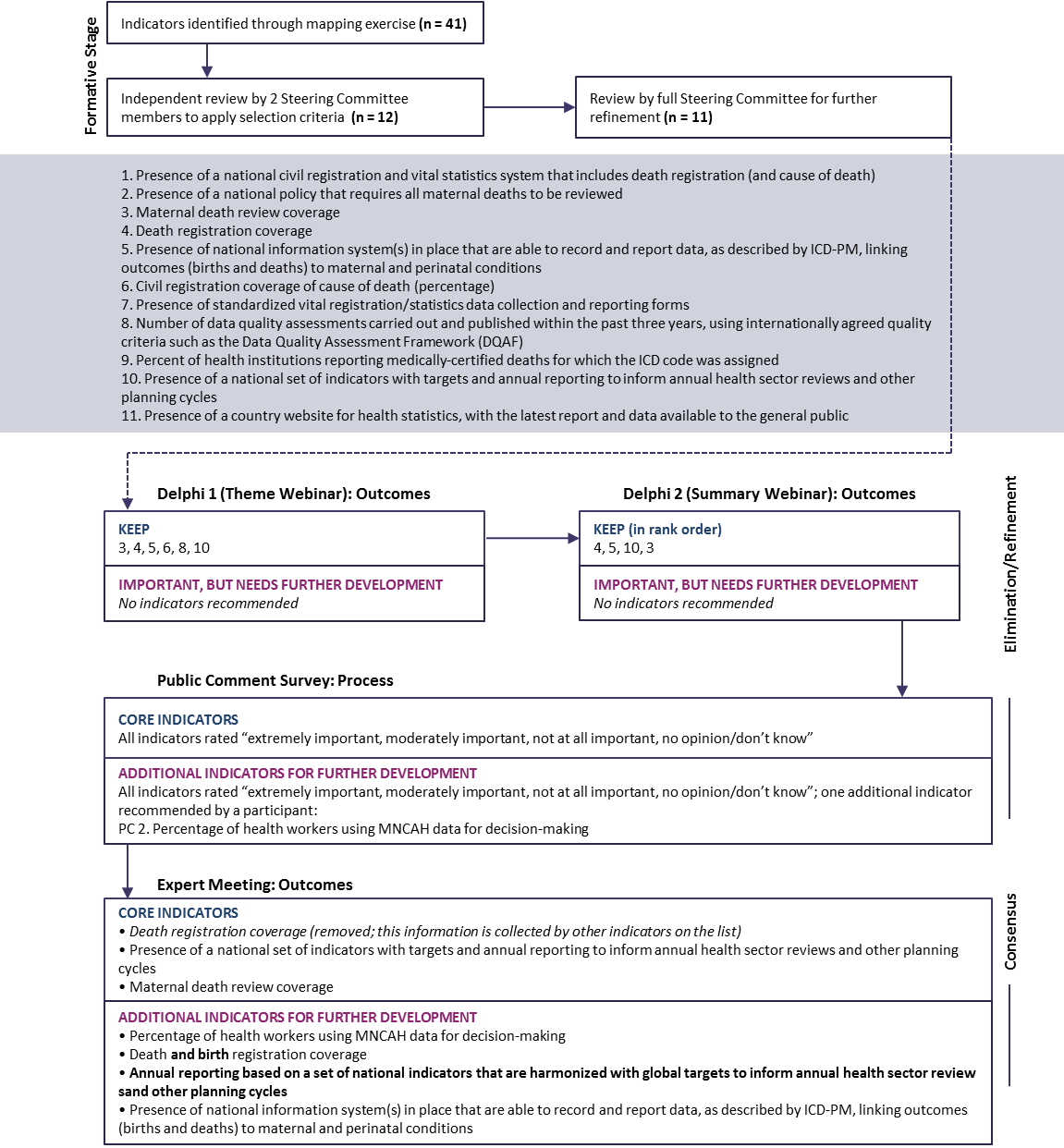
**

| **Theme 6: Prioritize adequate resources and effective health care financing** |
| --- |

**Priority Recommendations**

Adequate Resource Allocation

- Development partners and donors in the global community prioritize adequate and sustainable resources for maternal and newborn health
- Political leaders and financial decision makers in countries prioritize adequate and sustainable resources for maternal and newborn health
- There is adequate budgetary allocation for maternal and newborn health through specific, transparent budget lines
- In keeping with the Global Financing Facility (GFF), low- and middle-income countries allocate at least 3% of their gross domestic product to general government health expenditures of which at least 25% (and up to 50%) should be allocated to SRMNCAH
- There is national health care financing for UHC

Budget Tracking and Transparency

- There is budget transparency, assured through budget monitoring, analysis and advocacy, to allow public verification that policy commitments made are fulfilled and that policy decisions, including allocation of financial resources, are carried out on the basis of transparency, accountability, non-discrimination and participation
- Countries track and report total health expenditure by financing source, per capita, as recommended by CoIA
- Countries track and report total reproductive, maternal, newborn and child health expenditure by financing source, per capita, as recommended by CoIA

Effective Healthcare Financing Mechanisms

- There are innovative financing mechanisms and incentives to ensure equity, increase coverage and improve quality
- There is intersectoral collaboration between the health and financial sectors that includes both public and private national health care payers, ministries of finance, and private as well as bilateral global development partners and donors
- Countries monitor the full impact of financial incentives on maternal health outcomes

**Indicator Identification Process**

**
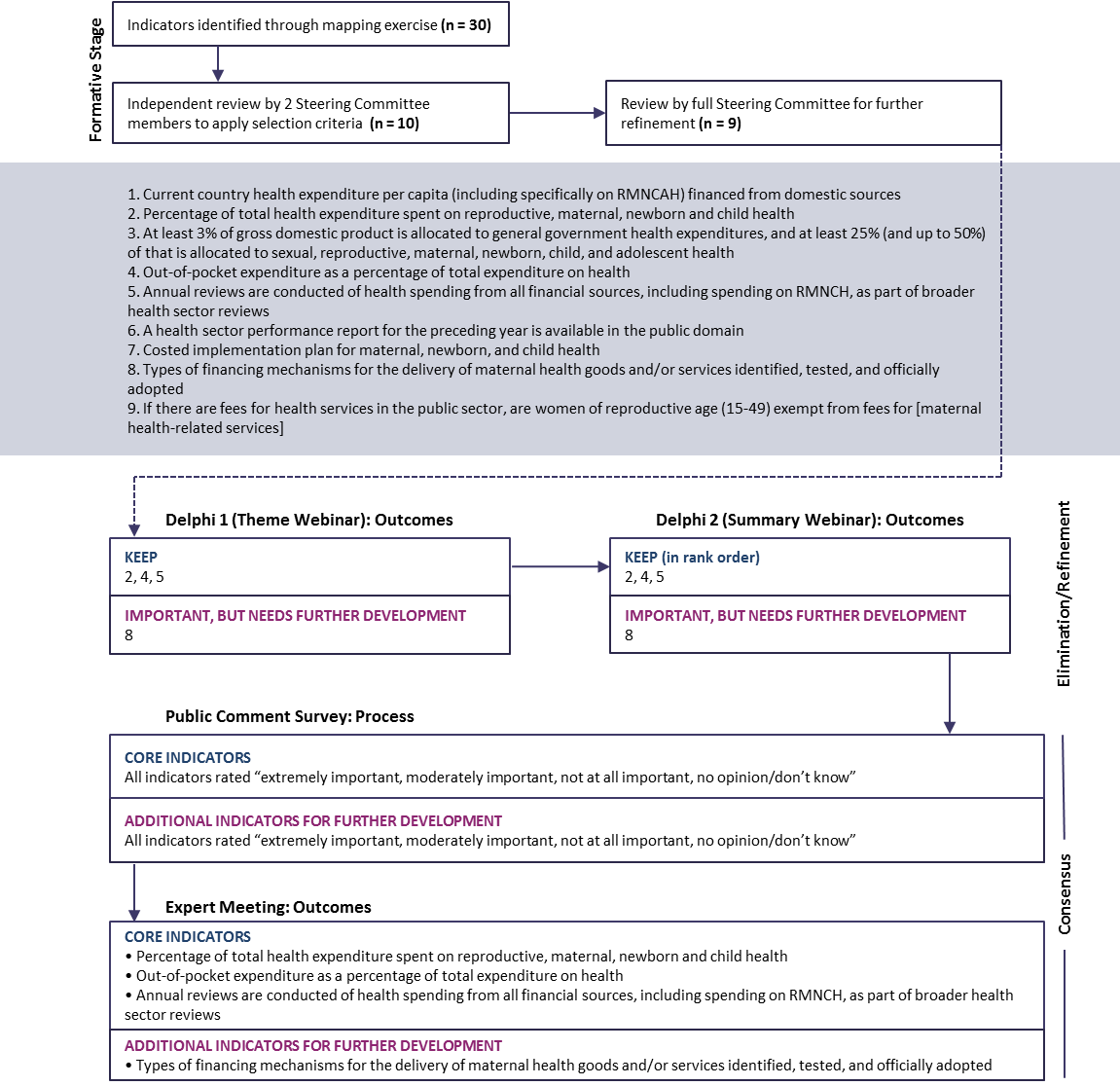
**

| **Theme 7: Address inequities in access to and quality of sexual, reproductive, maternal**  **and newborn health care** |
| --- |

**Priority Recommendations**

Improved Data to Address Disparities in SRMNCAH Care

- Disaggregated data are routinely collected and used to understand the determinants of inequities and to design, implement and monitor interventions to eliminate them
- Disparities are analyzed to determine how health system operations, planning and programming for maternal health, and service distribution result in inequitable health outcomes
- Governments and technical experts improve the availability and effective use of data on inequities and their effect on reproductive and maternal health
- Valid equity indicators are developed

Equitable Coverage and Quality of SRMNCAH Care

- High-quality primary and emergency SRMNCAH services reach vulnerable populations in all countries
- Program planners promote equitable coverage and equal access to sexual, reproductive, maternal and newborn health care services through better efforts to understand the unique challenges and needs of subpopulations within societies
- An adequate workforce is available to provide the full range of SRMNCAH care to all subpopulations
- Care that is offered to all populations is of comparably high quality

Supply-side Efforts to Address Inequities

- National planners identify and address barriers to access – financial, legal, gender, age, cultural, geographic or based on fear of disrespectful care
- Representatives from disadvantaged groups have a voice in processes to contextualize global best practices to yield effective, high-quality results in the context in which they are to be implemented and for all populations

**Indicator Identification Process**


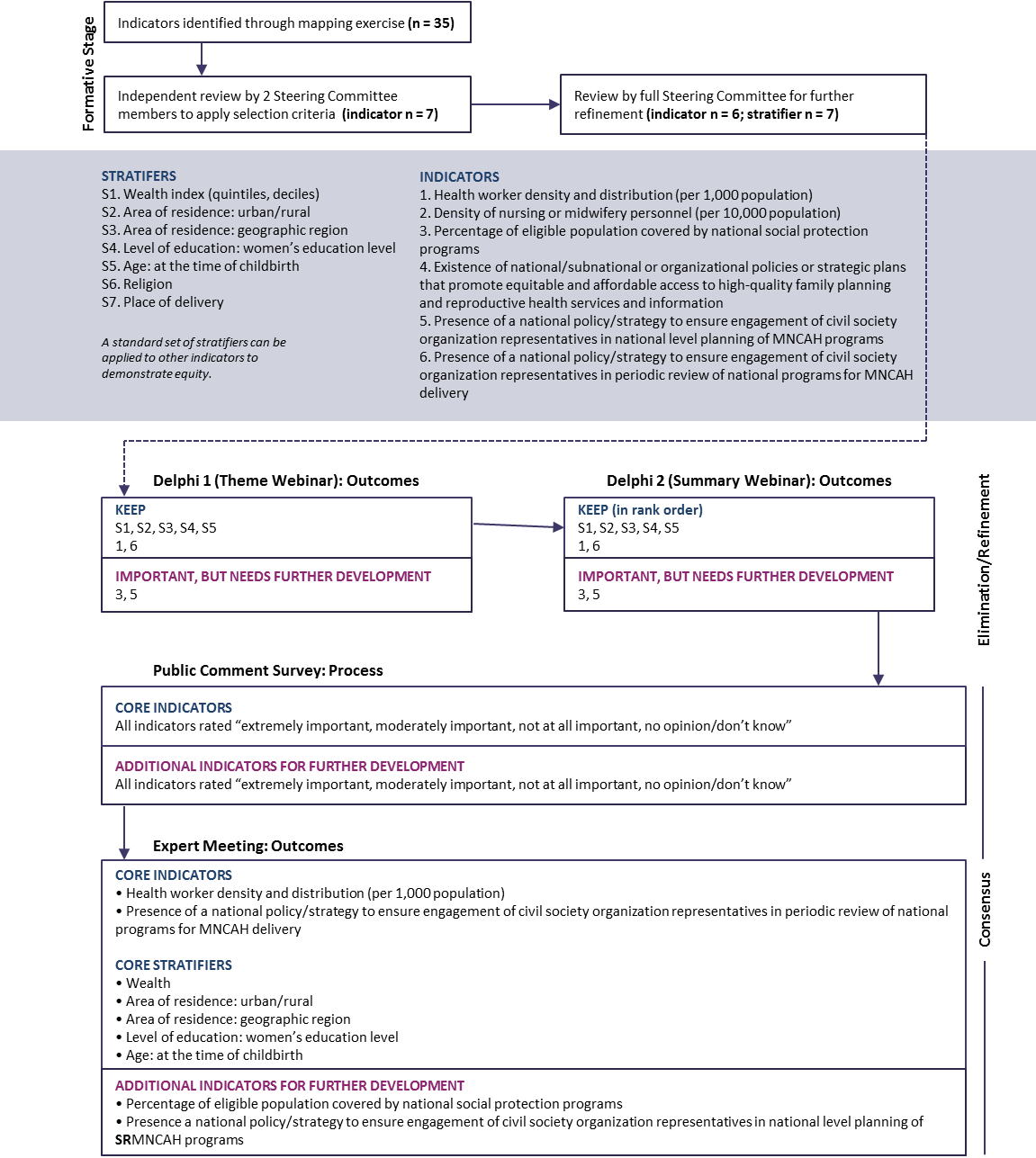


| **Theme 8: Ensure universal health coverage for comprehensive sexual, reproductive, maternal and newborn health care** |
| --- |

**Priority Recommendations**

Definition of an Essential Covered SRMNCAH Services Package

- There is a cost-effective national set of essential covered SRMNCAH services and commodities
- The priority covered package of essential covered services includes care during labor and childbirth, and inter-conception care, family planning, antenatal care and postpartum care
- There are standards for the indications and safe use of medical and surgical interventions, including caesarean section

Reaching all People with the Essential Covered Services Package

- There is access without discrimination, especially for the poor, vulnerable and marginalized segments of the population
- There is resource mobilization and effective service delivery to guarantee that the worst-off in the population are reached with the essential service package, based on an understanding of population demographics and planning for the appropriate number of human resources
- Monitoring and evaluation of UHC programs includes transparency and participatory mechanisms to include civil society in the decision making process to maximize ownership and promote accountability

Protecting People from Financial Hardship Due to the Cost of Health Care

- There are specific provisions to protect families accessing emergency obstetric care and emergency newborn care from financial catastrophe
- Governments institute publicly-funded insurance making essential services available to all without out-of-pocket expenditures
- Governments expand services through progressive mandatory prepayment and pooling of funds with exemptions for the poor, bolstered by a variety of financing mechanisms, to cover a larger benefits package

**Indicator Identification Process**


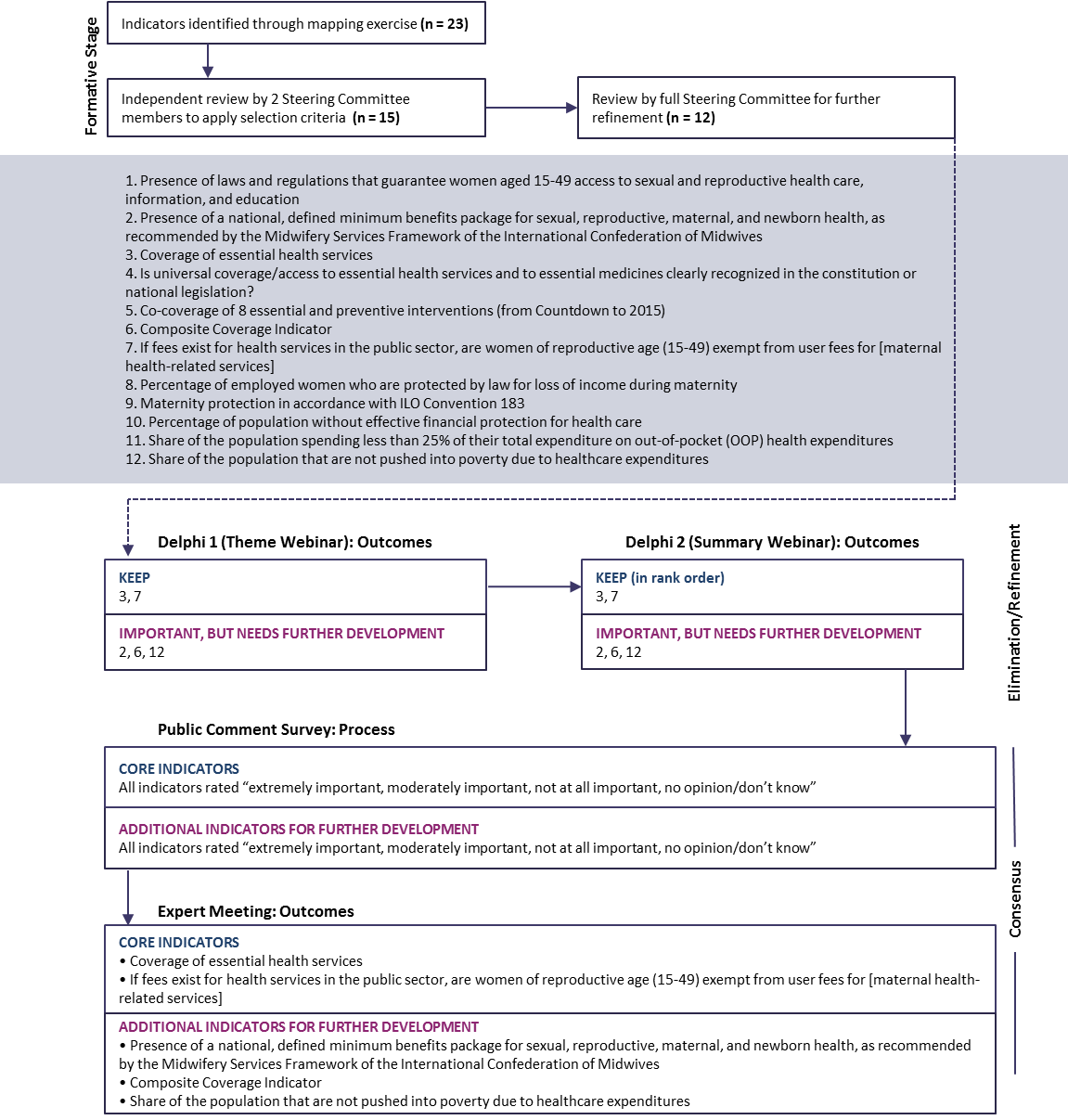


| **Theme 9: Address all causes of maternal mortality, reproductive and maternal morbidities, and related disabilities** |
| --- |

**Priority Recommendations**

Identify and Prioritize Country-Specific Maternal Health Risks

- Each country measures and tracks the most important causes of maternal deaths in its population
- Countries improve the quality of certification, registration, notification and review of causes of maternal death
- Countries demonstrate plans for tracking and treating maternal morbidities, using standard definitions and metrics
- Each country has a context-specific strategy for implementing effective interventions to address the most important causes of maternal death, key diseases and malnutrition, maternal morbidity and unmet need for family planning
- Prioritization for national plans is based on systematic analysis of context-specific determinants of risk and health systems capacity, including the human and financial resources available
- The obstetric transition model provides a foundation for country-specific analysis, adaptation based on local findings

Effective Strategies to Address Key Threats to Maternal Survival

- As MMR decreases, strategies for maternal mortality reduction address indirect causes
- Attention is taken to ensure that maternal causes of death that carry stigma, including abortion and HIV infection, are not underreported or misclassified
- There are effective strategies in place to prevent deaths following unsafe abortion
- There are effective strategies in place to prevent HIV-related deaths in pregnancy
- There are effective strategies in place to eliminate unmet need for family planning
- There are effective strategies in place to eliminate structural and social barriers that contribute to maternal death include delays in seeking, accessing and receiving appropriate treatment
- Effective interventions that address the most prevalent causes of death in the population are available at scale

Systematic Approaches to Program Effectiveness

- Each country’s framework is revisited at regular intervals to track progress, reassess the underlying assumptions and adjust the plan as needed
- There is evidence of intersectoral coordination to address all causes of maternal mortality, including WASH systems, roads and health care facilities, workforce planning and education for girls
- Each national strategy includes a systematic approach to implementing evidence-based standards, guidelines and protocols, and to monitoring and evaluating their outcomes through a large-scale process improvement initiative

**Indicator Identification Process**

**
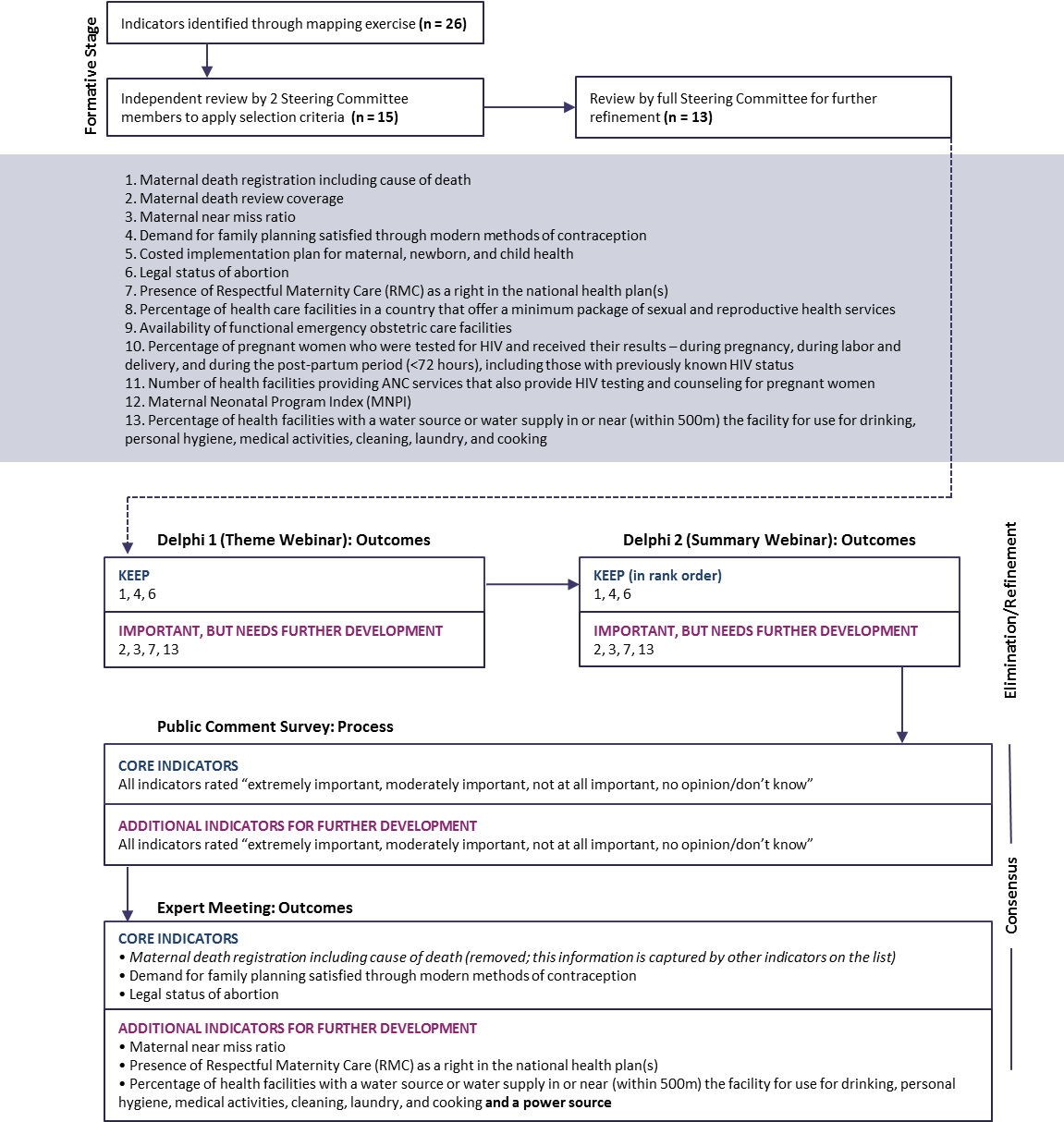
**

| **Theme 10: Strengthen health systems to respond to the needs and priorities of women and girls** |
| --- |

**Priority Recommendations**

Organization and Delivery of Maternal Health Services

- Service delivery includes expanded health promotion and preventative services
- Service delivery includes improved integration of all forms of care for women, newborns and adolescents, especially prevention, screening, and treatment for infectious and non-communicable diseases into routine SRMNCAH care
- Community-based primary care and effective referral systems are in place, ensuring seamless coordination across time, settings and disciplines, and between facilities
- There is universal coverage of essential maternal, newborn and family planning interventions that fall within the scope of midwifery practice

Workforce Management and Support

- Midwives, educated and regulated to international standards and working in well-equipped enabling environments, provide 87% of essential maternal and newborn health care services
- Governments provide appropriate regulatory support, pre-service and in-service training, and sufficient resources to deploy health care providers (midwives, doctors, and other skilled maternity care providers, including specialists) in adequate numbers to meet population needs
- Country-level workforce management ensures optimal recruitment, distribution and retention of health workers, enacts supportive supervision, and explores task shifting as needed to improve access to high-quality care

Essential Health Infrastructure and Amenities

- Essential health infrastructure and amenities are in place and functioning to the level of need, including an adequate number of beds and skilled providers for the population to be served, as well as reliable power and WASH, rooms or dividers that ensure privacy, communication and computer equipment with good connectivity and access to emergency transportation
- Essential commodities and appropriate technologies are reliably available at the point of service, based on considerations of equity and cost-effectiveness
- A functioning and user-friendly health information system is in place to assist in data collection, as well as communication and coordination between levels of care, and between providers and patients

Intersectoral Collaboration

- There is evidence of effective cooperation with other sectors (such as finance, education, energy, water and sanitation, nutrition, social services, mobile telecommunications technology and private health care services) to promote good reproductive and maternal health outcomes

**Indicator Identification Process**

**
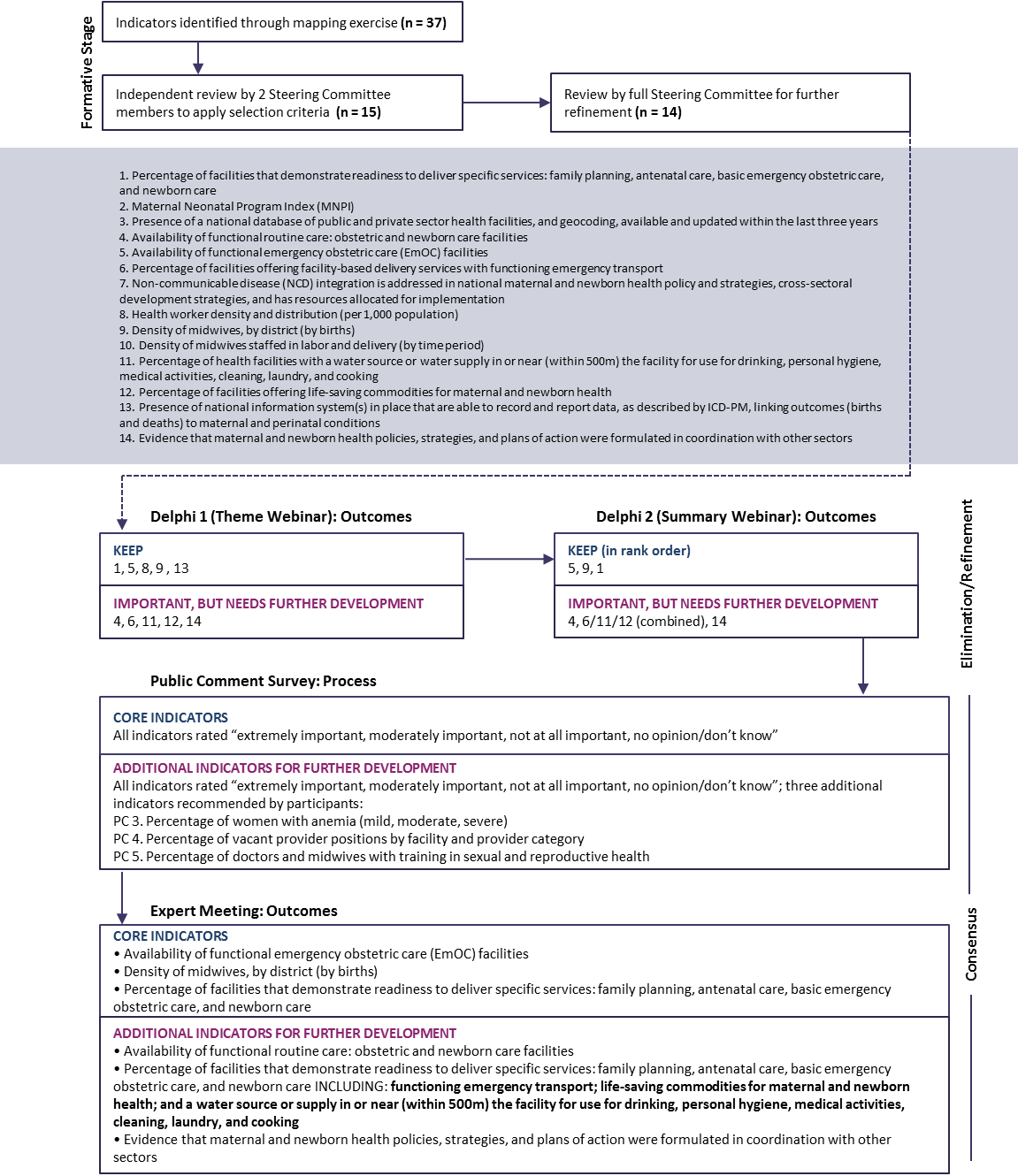
**

| **Theme 11: Ensure accountability in order to improve quality of care and equity** |
| --- |

**Priority Recommendations**

Data Tracking and Reporting for Accountability

- Countries track and measure progress towards EPMM and routinely report on it
- Countries strengthen civil registration systems that can provide reliable information on cause of death
- Countries build and strengthen national and subnational data collection through routine periodic data collection, increased measurement capacity, and informative monitoring and reporting
- National data registries collect data on the causes and conditions of every maternal death through confidential enquiries or maternal death surveillance and response (MDSR)
- National data registries collect data on cases of severe maternal morbidity through a near-miss reporting approach

Participation, Social Accountability, and Right to Remedy

- Citizens and civil society actors hold government and health system leaders to account for their commitments in the area of maternal and newborn health care delivery through social accountability mechanisms
- There are participatory mechanisms at every level of the health system, across public and private sectors
- Transparent and equitable legal frameworks ensure not only citizens’ rights to participation but also their right to remedy where appropriate

**Indicator Identification Process**

**
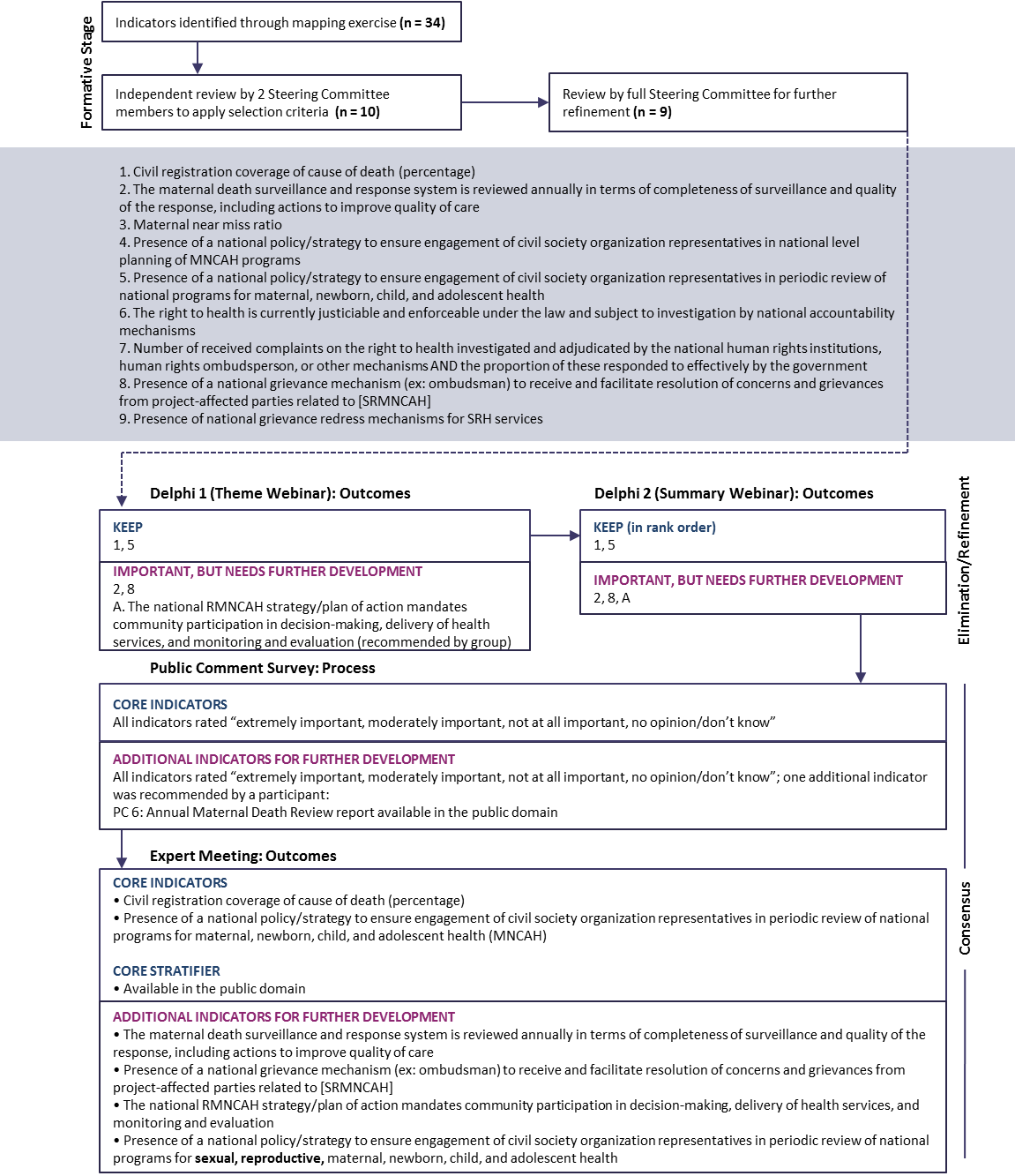
**
