# Supplementary material for: Ending preventable maternal mortality: phase II of a multi-step process to develop a monitoring framework, 2016–2030
Source: BMC Pregnancy Childbirth. 2018 Jun 25;18:258. doi: 10.1186/s12884-018-1763-8 (PMC6019318; doi:10.1186/s12884-018-1763-8)
Supplement: Supplementary file 2 — List of Participating Organizations. (DOCX 27 kb) [file 12884_2018_1763_MOESM2_ESM.docx]

Additional File 2. List of Participating Organizations

|  |  | Phase of Process | | | | |
| --- | --- | --- | --- | --- | --- | --- |
| Organization | Location | Steering Committee | Delphi 1 (Theme Webinar) | Delphi 2 (Summary Webinar) | Public Comment | Expert Meeting |
| **UN Agencies** | | | | | | |
| OHCHR |  |  | ✓ | ✓ | ✓ | ✓ |
| UNFPA |  | ✓ | ✓ | ✓ |  | ✓ |
| UNICEF |  |  | ✓ |  | ✓ | ✓ |
| WHO |  | ✓ | ✓ | ✓ |  | ✓ |
| World Bank |  |  | ✓ |  |  |  |
| **Programs** | | | | | | |
| American College of Nurse Midwives | USA |  |  |  | ✓ |  |
| African Health Budget Network | Tanzania |  |  |  |  | ✓ |
| Ariadne Labs | USA |  |  |  | ✓ |  |
| Averting Maternal Death and Disability (AMDD) | USA | ✓ | ✓ |  |  | ✓ |
| BankaBioLoo | India |  |  |  | ✓ |  |
| CARE | India |  |  |  | ✓ |  |
| Centre for Health and Social Justice | India |  | ✓ |  |  | ✓ |
| Center for Reproductive Rights | USA, Nigeria |  | ✓ |  |  |  |
| Catholic Medical Mission Board | Peru |  | ✓ |  |  | ✓ |
| CEPAL | Chile |  | ✓ |  |  |  |
| CHETNA | India |  |  |  | ✓ |  |
| Childbirth Survival International | USA |  |  |  | ✓ |  |
| Countdown to 2030 | USA, UK | ✓ | ✓ | ✓ |  | ✓ |
| Cultural Practice | USA |  | ✓ |  |  |  |
| Every Newborn Action Plan | UK |  | ✓ | ✓ |  | ✓ |
| Evidence 4 Action | UK |  | ✓ | ✓ |  | ✓ |
| FCI Program of MSH | USA |  | ✓ |  |  |  |
| FHI 360 | USA |  | ✓ |  |  |  |
| Guttmacher Institute | USA |  | ✓ | ✓ |  |  |
| HealthRight International | USA |  |  |  | ✓ |  |
| High-level Task Force for ICPD | USA |  | ✓ |  |  |  |
| icddr,b | Bangladesh |  | ✓ | ✓ |  | ✓ |
| ICF International | USA |  | ✓ |  |  | ✓ |
| INPPARES | Peru |  |  |  | ✓ |  |
| IPE Global | India |  |  |  | ✓ |  |
| Jhpiego | USA, Madagascar |  | ✓ |  | ✓ | ✓ |
| John Snow International | USA, Ethiopia, Ghana |  | ✓ | ✓ | ✓ |  |
| LAC Neonatal Alliance | Bolivia |  | ✓ |  |  |  |
| Management Sciences for Health | DRC, Kenya |  | ✓ |  | ✓ |  |
| Maternal and Child Survival Program | Madagascar, USA |  | ✓ |  | ✓ | ✓ |
| MCHIP | Zimbabwe |  |  |  | ✓ |  |
| MEASURE Evaluation | USA |  | ✓ |  |  |  |
| MHTF | USA | ✓ | ✓ | ✓ |  | ✓ |
| Navya Tarang Foundation | India |  |  |  | ✓ |  |
| Public Health Foundation of India | India |  |  |  | ✓ |  |
| Population Council | USA |  | ✓ |  | ✓ | ✓ |
| SDA Hospital | Ghana |  |  |  | ✓ |  |
| Save the Children | UK |  | ✓ |  | ✓ |  |
| Saving Newborn Lives | Bangladesh |  | ✓ |  | ✓ |  |
| SSQH-Nord | Haiti |  |  |  | ✓ |  |
| White Ribbon Alliance | USA | ✓ |  |  |  | ✓ |
| **Universities or Research Institutes** | | | | | | |
| Dublin City University | Ireland |  |  |  | ✓ |  |
| Federal University of Pelotas | Brazil |  | ✓ | ✓ |  |  |
| Georgetown Law | USA |  | ✓ |  |  |  |
| Gombe State University | Nigeria |  |  |  | ✓ |  |
| International Development Research Centre | Canada |  |  |  | ✓ |  |
| Ifakara Health Institute | Tanzania |  | ✓ |  |  | ✓ |
| Instituto Universitario de Lisboa | Portugal |  |  |  | ✓ |  |
| Karolinska Institut | Sweden |  | ✓ |  |  |  |
| Liverpool School of Tropical Medicine | UK |  |  |  | ✓ |  |
| London School of Hygiene and Tropical Medicine | UK |  | ✓ |  | ✓ | ✓ |
| Makarere University | Uganda |  | ✓ |  |  |  |
| Nell Hodgson Woodruff School of Nursing, Emory University | USA |  | ✓ | ✓ |  |  |
| Nossal Institute for Global Health, University of Melbourne | Australia |  |  |  | ✓ |  |
| University of British Columbia | Canada |  |  |  | ✓ |  |
| University of Dundee | UK (Scotland) |  | ✓ |  |  |  |
| University of Essex | UK |  | ✓ |  |  |  |
| University of Southampton | UK |  | ✓ |  |  | ✓ |
| University of West Indies, Mona | Jamaica |  | ✓ |  |  | ✓ |
| University Research Co. | Ecuador |  | ✓ |  | ✓ | ✓ |
| Walchand College | India |  |  |  | ✓ |  |
| **Hospitals** | | | | | | |
| Nayati Multi Super Specialty Hospital | India |  |  |  | ✓ |  |
| SDA Hospital | Ghana |  |  |  | ✓ |  |
| St. John of God, Subaico | Australia |  |  |  | ✓ |  |
| **Ministries of Health** | | | | | | |
| Ministry of Health, Ethiopia |  |  | ✓ |  |  | ✓ |
| Ministry of Health, Malawi |  |  | ✓ |  |  | ✓ |
| Ministry of Health, Tanzania |  |  | ✓ |  |  |  |
| **Donors** | | | | | | |
| Children’s Investment Fund Foundation | UK |  | ✓ |  |  |  |
| Bill & Melinda Gates Foundation | USA |  |  |  |  | ✓ |
| USAID | USA, India | ✓ | ✓ | ✓ |  | ✓ |
