# Supplementary material for: Ending preventable maternal mortality: phase II of a multi-step process to develop a monitoring framework, 2016–2030
Source: BMC Pregnancy Childbirth. 2018 Jun 25;18:258. doi: 10.1186/s12884-018-1763-8 (PMC6019318; doi:10.1186/s12884-018-1763-8)
Supplement: Supplementary file 3 — EPMM Phase II Indicators and Meta-Data. (PDF 107 kb) [file 12884_2018_1763_MOESM3_ESM.pdf]

Additional File 3. EPMM Phase II Indicators and Meta-data

This display groups the EPMM Phase II indicators by type - Categorical and Percentage/Proportional - in order to make the table headings as simple and consistent as possible.

Categorical Indicators

Indicator	Present	Not Present	Partial	Data Source	Reference
**Presence of laws and regulations that guarantee women aged 15-49 access to sexual and reproductive health care, information, and education**<br>Laws and regulations guaranteeing access include:<br>• Access to sexual and reproductive health services without third-party authorization<br>• Access to sexual and reproductive health services without restrictions in regards to age or marital status<br>• Adolescent access to sexual and reproductive health information and education				Self-reported by government	Modified from SDG Meta-data for Goal 5
**Whether or not legal frameworks are in place to promote, enforce, and monitor equality and non-discrimination on the basis of sex**				World Bank's Women, Business, and the Law Survey; OECD Social Institutions and Gender Index (DHS, MICS, Administrative data)	SDG Meta-data for Goal 5
**Presence of protocols/policies on combined care of mother and baby, immediate breastfeeding, and observations of care**<br>Staff encourage skin-to-skin contact<br>• Mothers should be able to hold newborns immediately after birth and breastfeed their babies as soon as possible after birth<br>• The facility provides combined care for mother and baby and space should accommodate mother/newborn pairs after delivery<br>• All staff are trained regularly on newborn resuscitation<br>• Posters/signs for mothers/newborns are in local languages and heavily graphic				Facility surveys	FIGO/ICM/WRA/IPA/WHO Mother and Baby Friendly facility criteria
**Maternity protection in accordance with ILO Convention 183**	ILO Convention 183 ratified (maternity leave of at least 14 weeks with cash benefits of previous earnings paid by social security or public funds)	No ratification of any maternal protection convention	ILO Convention 183 not ratified but previous maternity convention ratified (maternity leave of at least 12 weeks with cash benefits of previous earnings paid by social security or public funds	National legislation	Countdown to 2015
**International Code of Marketing of Breastmilk Substitutes**	All provisions stipulated in ICMBS adopted in legislation	No legislation and no voluntary agreements adopted in relation to the ICMBS	Voluntary agreements or some provisions stipulated in ICMBS adopted in legislation	WHO	Countdown to 2015
**Costed implementation plan for maternal, newborn, and child health**	Costed plan or plans to scale up maternal, newborn and child health interventions available at national level	No costed implementation plan for maternal, newborn and child health available	Costed plan available for either maternal and newborn health or child health	WHO, Global Health Atlas	Countdown to 2015
**Midwives are authorized to deliver basic emergency obstetric and newborn care**<br>Eight functions of basic emergency obstetric care:<br>• Parental antibiotics, oxytocics, anticonvulsants<br>• Manual removal of placenta<br>• Removal of retained products of conception<br>• Assisted vaginal delivery<br>• Newborn resuscitation	Midwives are authorized for all tasks	Midwives are not authorized for any of these tasks	Midwives are authorized for some of these tasks	WHO MCA Department special data compilation	Countdown to 2015
**Legal status of abortion**<br>*The critiera for ranking this indicator includes:*<br>*I = to save a woman's life*<br>*II = to preserve physical health and above*<br>*III = to preserve mental health and above*<br>*IV = for economic and social reason and the above*<br>*V = on request and above*<br>*R = in case of rape or incest*<br>*F = in case of fetal impairment*<br>*— = data are not available*				Country data and policies	Countdown to 2015

**Presence of a national set of indicators with targets and annual reporting to inform annual health sector reviews and other planning cycles**				National health sector reports	WHO, Health Information Systems Performance Index
**Annual reviews are conducted of health spending from all financial sources, including spending on RMNCH, as part of broader health sector reviews**					CoIA iERG recommendation
**Presence of a national policy/strategy to ensure engagement of civil society organization representatives in periodic review of national programs for MNCAH delivery**	Yes, for all RMNCH programs	Yes, in selected components of RMNCH program	Civil society organizations not engaged	WHO MNCAH Policy Survey 2016	WHO MNCAH Policy Survey, 2016 (not publicly available at this time; more information about the survey available here)
**If fees exist for health services in the public sector, are women of reproductive age (15-49) exempt from user fees for the following services:**<br>**• Family planning**<br>**• Antenatal care**<br>**• Childbirth (normal delivery)**<br>**• Post-natal care for mother**<br>**• Caesarean section**<br>**• Insecticide-treated bednets**<br>**• Pharmaceutical products and/or other medical supplies if required for treatment or delivery**<br>**• Immunization services (TT, Rubella)**	Yes, women are exempt from user fees	No, women are not exempt from user fees	Yes, women are exempt by type of service	WHO MNCAH Policy Survey 2016	WHO MNCAH Policy Survey, 2016 (not available publicly at this time; more information on the survey available here)

Percentage/Proportional Indicators

Indicator	Numerator	Denominator	Disaggregator(s)	Data Source	Reference
**Gender Parity Index**	Female gross enrollment rate	Male gross enrollment rate		Administrative data	SDG Meta-data for Goal 4
**Proportion of women aged 15-49 who make their own informed and empowered decisions regarding sexual relations, contraceptive use, and reproductive health care**<br>The numerator includes women who answer "yes" to the following three questions:<br>• Whether a woman can say no to her husband/partner<br>• Whether using or not using contraception has been mainly the woman's decision<br>• Whether a woman can make a decision about sexual and reproductive health herself if she does not want to have sexual intercourse	Number of women and girls (ages 15-49) who make decisions about their own sexual and reproductive health and reproductive rights	Total number of women and girls (ages 15-49)	Age, location, economic quintile, education, marital status, disability	DHS; MICS; household surveys	SDG Meta-data for Goal 5
**Geographic distribution of facilities that provide basic and comprehensive emergency obstetric care (EmOC)**	Number of facilities in the area providing comprehensive EmOC	Population of area divided by 500,000		Facility surveys with medical records or service statistics	WHO/UNFPA/UNICEF/AMDD (more information at MEASURE FP and RH Indicators Database)
**Maternal death review coverage**	Number of health facility maternal deaths that were reviewed	All maternal deaths in facilities	Facilities, major administrative regions	Facility reports	WHO Core Health Indicators
**Percentage of total health expenditure spent on reproductive, maternal, newborn, and child health**	Total RMNCH expenditure	Total population	Funding source	Government Ministry reports, NHA data, public expenditure reviews	Adapted from CoIA Recommendation
**Out-of-pocket expenditure as a percentage of total expenditure on health**	Out-of-pocket expenditure on health	Total expenditure on health		Global Health Expenditure Database	Countdown to 2015
**Health worker density and distribution (per 1,000 population)**	Number health workers by cadre	Total population	Cadre (generalist or specialist medical practitioners, nursing and midwifery professionals, traditional and complementary medicine professionals, others)	Health worker registry	Metadata for SDG Goal 3 (pg 47), and WHO Core Health Indicators
**Coverage of essential health services**	Number of people receiving the intervention	Number of people who need the intervention	Sex, age, SES, geography, type of intervention	Household surveys, facility data	Proposed indicator, meta-data for SDG Goal 3 (pg 40)
**Demand for family planning satisfied through modern methods of contraception**	Women who are married/in a union and currently using any method of contraception	Women who are married/in a union and who are currently using any method of contraception or who are fecund, not using any method of contraception but wanting to space their next birth or stop childbearing altogether	Wealth, age, education, residence	MICS, DHS, RHS, other national surveys	Modified from Countdown to 2015

**Availability of functional emergency obstetric care (EmOC) facilities**	Number of obstetric care facilities that provided EmONC signal functions in the last three months	Per 500,000 population	Subnational facility level	Health facility surveys, routine facility monitoring, census or other population data source	WHO/UNICEF/UNFPA/AMDD Monitoring Emergency Obstetric Care: A Handbook (pg 10)
**Density of midwives, by district (by births)**	Number of midwives in a district	All births in a district		Surveys	WHO Consultation on Improving Measurement of Quality of MNCH in Facilities (pg 30)
**Percentage of facilities that demonstrate readiness to deliver specific services: family planning, antenatal care, basic emergency obstetric care, and newborn care**<br>ANC tracer items include:<br>Staff and Training<br>• Guidelines on ANC<br>• Staff trained in ANC<br>Equipment<br>• Blood pressure machine<br>• Stethoscope<br>Diagnostic<br>• Hemoglobin<br>• Urine protein<br>Medicines and Commodities<br>• Iron tablets<br>• Folic acid tablets<br>• Tetanus toxoid	Number of tracer items for a service present and functioning	Total number of tracer items for a service		Health facility assessments	WHO "Monitoring the Building Blocks of Health Systems Handbook" (pg 18)
**Civil registration coverage of cause of death (percentage)**	Total number of deaths that have been registered with cause-of-death information in the vital registration system for a country-year	Total estimated deaths for that year for the national population		Civil registration system	WHO Indicator and Measurement Registry

Standard Stratifiers

Stratifier	Area of Measurement	Reference
Wealth	Equity	Countdown to 2030
Area of residence: urban/rural	Equity	Countdown to 2030
Area of residence: geographic region	Equity	Countdown to 2030
Level of education: women's education level	Equity	Countdown to 2030
Age	Equity	Countdown to 2030
"Available in the public domain"	Transparency	
