# Supplementary material for: Ending preventable maternal mortality: phase II of a multi-step process to develop a monitoring framework, 2016–2030
Source: BMC Pregnancy Childbirth. 2018 Jun 25;18:258. doi: 10.1186/s12884-018-1763-8 (PMC6019318; doi:10.1186/s12884-018-1763-8)
Supplement: Supplementary file 4 — Phase II Indicators by Maternal Health Topic Area. (DOCX 33 kb) [file 12884_2018_1763_MOESM4_ESM.docx]

Additional File 4. Phase II Indicators by Maternal Health Topic Area

| **Indicator** | **Health System Strengthening** | **Human Rights** | **Universal Health Coverage** | **Empowering Girls & Women** | **Improving Metrics & Measurement** |
| --- | --- | --- | --- | --- | --- |
| Presence of laws and regulations that guarantee women aged 15-49 access to sexual and reproductive health care, information, and education | ✓ | ✓ |  | ✓ |  |
| Gender Parity Index (GPI) |  | ✓ |  | ✓ |  |
| Whether or not legal frameworks are in place to promote, enforce, and monitor equality and non-discrimination on the basis of sex | ✓ | ✓ |  | ✓ |  |
| Presence of protocols/policies on combined care of mother and baby, immediate breastfeeding, and observations of care | ✓ |  |  | ✓ |  |
| Maternity protection in accordance with ILO Convention 183 | ✓ | ✓ |  | ✓ |  |
| International Code of Marketing of Breastmilk Substitutes | ✓ |  |  |  |  |
| Costed implementation plan for maternal, newborn, and child health | ✓ | ✓ | ✓ |  |  |
| Midwives are authorized to deliver basic emergency obstetric and newborn care | ✓ |  |  |  |  |
| Legal status of abortion | ✓ | ✓ |  | ✓ |  |
| Proportion of women aged 15-49 who make their own informed decisions regarding sexual relations, contraceptive use, and reproductive health care |  | ✓ |  | ✓ |  |
| Geographic distribution of facilities that provide basic and comprehensive emergency obstetric care (EmOC) | ✓ | ✓ |  |  |  |
| Presence of a national set of indicators with targets and annual report to inform annual health sector reviews and other planning cycles | ✓ |  | ✓ |  | ✓ |
| Maternal death review coverage | ✓ |  | ✓ |  | ✓ |
| Percentage of total health expenditure spent on reproductive, maternal, newborn, and child health | ✓ | ✓ | ✓ |  |  |
| Out-of-pocket expenditure as a percentage of total expenditure on health | ✓ | ✓ | ✓ |  |  |
| Annual reviews are conducted of health spending from all financial sources, including spending on RMNCH, as part of broader health sector reviews | ✓ | ✓ | ✓ |  | ✓ |
| Health worker density and distribution (per 1,000 population) | ✓ |  |  |  |  |
| Coverage of essential health services | ✓ | ✓ | ✓ |  |  |
| If fees exist for health services in the public sector, are women of reproductive age (15-49) exempt from user fees for [MH-related health] services | ✓ | ✓ | ✓ | ✓ |  |
| Demand for family planning satisfied through modern methods of contraception | ✓ | ✓ |  | ✓ |  |
| Availability of functional emergency obstetric care (emOC) facilities | ✓ | ✓ |  |  |  |
| Density of midwives, by district (by births) | ✓ |  |  |  |  |
| Percentage of facilities that demonstrate readiness to deliver specific services: family planning, antenatal care, basic emergency obstetric care, and newborn care | ✓ |  |  |  |  |
| Civil registration coverage of cause of death (percentage) | ✓ |  |  |  | ✓ |
| Presence of a national policy/strategy to ensure engagement of civil society organization representatives in periodic review of national programs for maternal, newborn, child, and adolescent health (MNCAH) |  | ✓ |  | ✓ |  |
| **Stratifier: Equity** |  |  |  |  |  |
| Wealth |  | ✓ |  |  |  |
| Area of residence: urban/rural |  | ✓ |  |  |  |
| Area of residence: geographic region |  | ✓ |  |  |  |
| Level of education: women's education level |  | ✓ |  |  |  |
| Age |  | ✓ |  |  |  |
| **Stratifier: Transparency** |  |  |  |  |  |
| “Available in the public domain” |  | ✓ |  |  |  |
|  |  |  |  |  |  |
